# Targeted Cancer Therapy‐on‐A‐Chip

**DOI:** 10.1002/adhm.202400833

**Published:** 2024-08-05

**Authors:** Heba Abed, Remya Radha, Shabana Anjum, Vinod Paul, Nour AlSawaftah, William G. Pitt, Nureddin Ashammakhi, Ghaleb A. Husseini

**Affiliations:** ^1^ Department of Chemical and Biological Engineering American University of Sharjah Sharjah UAE; ^2^ Materials Science and Engineering PhD program College of Arts and Sciences American University of Sharjah Sharjah UAE; ^3^ Department of Chemical Engineering Brigham Young University Provo UT 84602 USA; ^4^ Institute for Quantitative Health Science and Engineering (IQ) and Department of Biomedical Engineering (BME) Michigan State University East Lansing MI 48824 USA; ^5^ Department of Bioengineering University of California, Los Angeles Los Angeles CA 90095‐1600 USA

**Keywords:** 3D culture, chemotherapy, drug delivery, microfluidic, organ‐on‐a‐chip, targeting

## Abstract

Targeted cancer therapy (TCT) is gaining increased interest because it reduces the risks of adverse side effects by specifically treating tumor cells. TCT testing has traditionally been performed using two‐dimensional (2D) cell culture and animal studies. Organ‐on‐a‐chip (OoC) platforms have been developed to recapitulate cancer in vitro, as cancer‐on‐a‐chip (CoC), and used for chemotherapeutics development and testing. This review explores the use of CoCs to both develop and test TCTs, with a focus on three main aspects, the use of CoCs to identify target biomarkers for TCT development, the use of CoCs to test free, un‐encapsulated TCTs, and the use of CoCs to test encapsulated TCTs. Despite current challenges such as system scaling, and testing externally triggered TCTs, TCToC shows a promising future to serve as a supportive, pre‐clinical platform to expedite TCT development and bench‐to‐bedside translation.

## Graphical abstract

Targeted cancer therapy (TCT) minimizes side effects by specifically treating tumor cells. Traditionally tested with 2D cell cultures and animal studies, TCT now utilizes cancer‐on‐a‐chip (CoC) platforms for in vitro testing. This review examines CoCs in developing and testing TCTs, identifying biomarkers, and evaluating both free and encapsulated TCTs, highlighting CoC's potential in pre‐clinical applications despite current challenges.

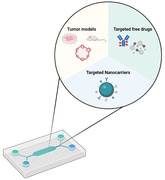

## 1 Introduction

Chemotherapy is one of the most prevalent treatments for cancer patients.^[^ ^1^ ^]^ It is used as a primary treatment for patients with advanced metastatic cancers with no alternative treatment options, a neoadjuvant treatment prior to surgery, and an adjuvant treatment after surgery.^[^ ^1^ ^]^ However, due to its low specificity, conventional chemotherapy is associated with unwanted toxic effects leading to anemia, infection, gastrointestinal tract upset, and other problems that affect the quality of life of patients.^[^ ^2^, ^3^, ^4^, ^5^, ^6^, ^7^, ^8^, ^9^, ^10^ ^]^ To lower the risk of developing side effects, research focused on developing specific cancer therapies^[^ ^11^, ^12^, ^13^, ^14^ ^]^ such as targeted cancer therapy (TCT).^[^ ^11^, ^13^, ^15^, ^16^, ^17^ ^]^ Because of their high specificity^[^ ^18^ ^]^ targeted therapies have already been shown to reduce neutropenia,^[^ ^19^ ^]^ mitigate off‐target organ drug accumulation, and overcome multi‐drug resistance (MDR).^[^ ^17^, ^20^, ^21^, ^22^ ^]^ Moreover, a significant advantage of TCTs is the ability to use higher doses of cytotoxic agents to achieve more effective treatment.^[^ ^16^ ^]^

To test TCT, two‐dimensional (2D) cell culture^[^ ^23^, ^24^, ^25^, ^26^ ^]^ and experimental animals^[^ ^27^, ^28^, ^29^, ^30^, ^31^ ^]^ have conventionally been used. In addition, the use of three‐dimensional (3D) cultures, such as spheroids,^[^ ^32^, ^33^, ^34^, ^35^ ^]^ organoids,^[^ ^32^, ^33^, ^36^, ^37^, ^38^, ^39^, ^40^ ^]^ tissue engineering,^[^ ^41^, ^42^, ^43^, ^44^ ^]^ and 3D bioprinting^[^ ^45^, ^46^, ^47^, ^48^, ^49^ ^]^ constructs, have recently been explored as an alternative approach.^[^ ^50^, ^51^, ^52^, ^53^, ^54^ ^]^ However, 2D cultures cannot reproduce the in vivo environment.^[^ ^23^, ^55^, ^56^ ^]^ Organoids and spheroids are static models and suffer from poor reproducibility.^[^ ^23^, ^24^, ^25^, ^26^, ^29^, ^50^, ^57^, ^58^, ^59^ ^]^ Tissue engineering is limited in providing precise control of cell placement^[^ ^60^ ^]^ and 3D bioprinting methods lack the representation of many aspects of the in vivo environment, such as the flow and biomimetic tissue organization.^[^ ^61^ ^]^ Experimental animals innately differ from humans and inaccurately represent human responses.^[^ ^57^, ^58^ ^]^ Therefore, the search for more efficient and biomimetic alternative models to study targeting, test TCT, and accelerate bench‐to‐bedside translation has been pursued.

Among the emerging alternative technologies for testing and developing TCTs are organ‐on‐a‐chip (OoC)‐based models.^[^ ^62^, ^63^, ^64^ ^]^ OoCs can recapitulate human tissues and organs on a smaller scale in a microfluidic chip device, including the dynamic processes seen in organs and tissues.^[^ ^62^, ^64^, ^65^ ^]^ Using OoC, human cancer cells can be cultured in the chip microchannels^[^ ^66^ ^]^ and dynamic flow conditions can be incorporated^[^ ^67^, ^68^, ^69^, ^70^, ^71^ ^]^ to produce a biomimetic cancer‐on‐a‐chip (CoC) model.^[^ ^66^, ^72^, ^73^ ^]^ Patient‐derived cells can also be used to develop more personalized therapies.^[^ ^74^, ^75^, ^76^ ^]^ Such CoC platforms have the potential to complement animal experiments and reduce the number of animals used. They can also potentially be used to plan clinical trials or perform clinical‐trials‐on‐a‐chip.^[^ ^77^, ^78^ ^]^ Artificial intelligence (AI) can also be incorporated to handle vast volumes of data resulting from numerous investigations on the chip, further expediting the drug discovery process.^[^ ^79^ ^]^

This niche research area is rapidly growing, and several reports on CoC models have been explored for screening chemotherapeutic agents for TCT to better understand their side effects and evaluate their efficacy.^[^ ^67^, ^69^, ^71^, ^80^, ^81^, ^82^, ^83^, ^84^, ^85^, ^86^, ^87^, ^88^, ^89^, ^90^, ^91^, ^92^, ^93^, ^94^, ^95^, ^96^, ^97^, ^98^, ^99^, ^100^, ^101^, ^102^ ^]^ Although there are excellent reviews on OoCs^[^ ^75^, ^103^, ^104^, ^105^, ^106^ ^]^ and CoC design and use,^[^ ^66^, ^72^, ^73^, ^76^, ^79^, ^107^, ^108^ ^]^ none is focused on the use of CoCs for studying and testing targeted therapy. Because CoCs are expected to develop into an important alternative platform for developing targeted chemotherapeutics, a review of this subject is essential to bridge this gap in the literature and help us develop important research directions.

**Figure 1:**
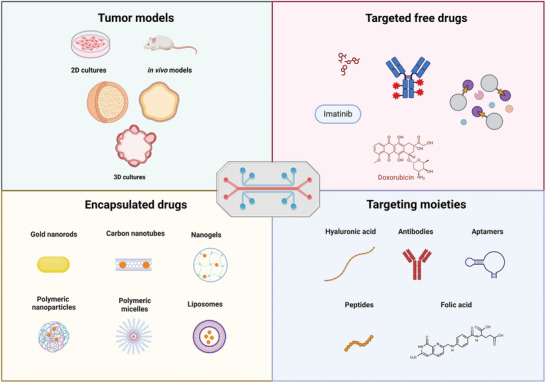
Exploring targeted cancer therapy‐on‐a‐chip (Created using BioRender.com).

Therefore, this review was developed to capture advancing frontiers and discuss emerging ideas, approaches, and potential applications of targeted cancer therapy‐on‐a‐chip (TCToC). In addition, current challenges facing the use of CoCs for studying targeting are explored, and future research directions are highlighted in **Figure** **1**.

## 2 Targeting Methods

Targeting methods used to design and develop targeted chemotherapies are plentiful and can be classified into three main types: passive, active (biological), and triggered.^[^ ^109^, ^110^, ^111^ ^]^

### 2.1 Passive Targeting

Passive targeting utilizes the leaky vasculature of solid tumors and the relatively loose cell‐to‐cell junctions to trap and accumulate therapeutics (and their carriers) of adequate size at the tumor site. This is referred to as the enhanced permeability and retention (EPR) effect.^[^ ^111^, ^112^ ^]^ Nanoparticle (NP) size, shape, stiffness, and surface properties, including surface charge, are all critical factors that influence accumulation at the tumor site by the EPR effect.^[^ ^113^ ^]^ Thus, these variables are essential when designing targeted chemotherapeutics utilizing passive targeting.

### 2.2 Active Targeting

In active or biological targeting, chemotherapeutic agents are modified with specific biomolecules that actively bind to certain molecular sites on cells, called receptors, and other targets characteristic of cancerous cells.^[^ ^114^, ^115^ ^]^ This method requires the receptor or target to be overly expressed in cancer cells but not normal cells, allowing for selective targeting.^[^ ^116^ ^]^ A wide range of biological moieties and ligands have been used to modify anti‐cancer agents and induce biological targeting, including proteins,^[^ ^117^ ^]^ peptides,^[^ ^118^, ^119^, ^120^ ^]^ antibodies,^[^ ^121^, ^122^, ^123^ ^]^ carbohydrates,^[^ ^124^, ^125^, ^126^, ^127^, ^128^ ^]^ aptamers,^[^ ^129^, ^130^ ^]^ and small molecules.^[^ ^18^, ^131^, ^132^ ^]^ These ligands can either be directly conjugated to the chemotherapeutic drug or to a carrier that can encapsulate an anti‐cancer drug and deliver it to cancer cells, such as liposomes, micelles, nanogels, etc.^[^ ^115^, ^116^ ^]^

### 2.3 Triggered Targeting

**Figure 2:**
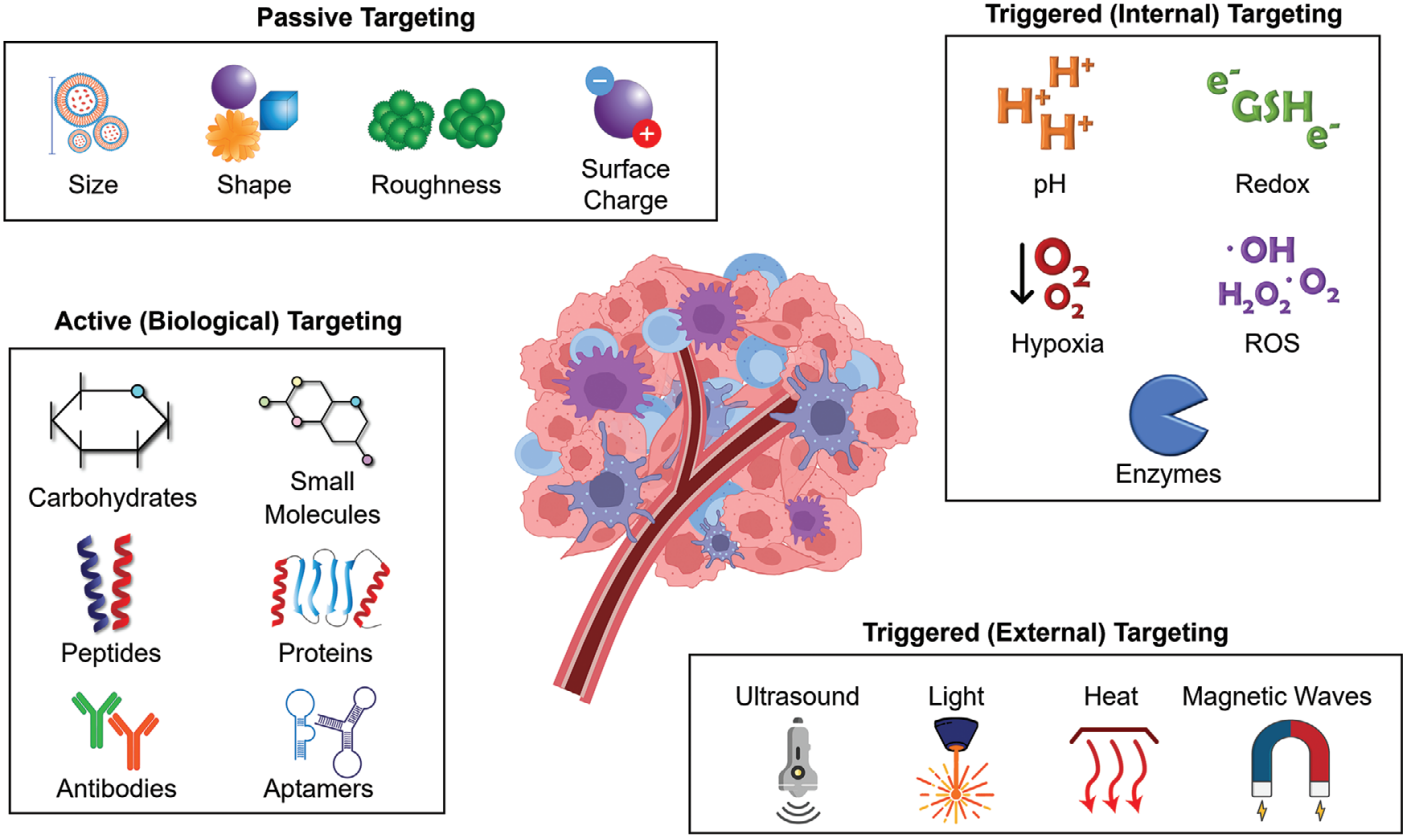
Summary of Tumor Targeting Methods. GSH: glutathione, ROS: reactive oxygen species.

Triggered targeting entails the use of a stimulus, intrinsic or extrinsic, to trigger drug activation or its release from a nanocarrier. Internal stimuli include pH,^[^ ^133^, ^134^, ^135^, ^136^ ^]^ enzymes,^[^ ^135^, ^137^, ^138^ ^]^ hypoxia,^[^ ^139^ ^]^ reactive oxygen species (ROS),^[^ ^140^, ^141^ ^]^ and redox (via the reductive environment of tumors),^[^ ^142^, ^143^ ^]^ all of which are unique internal characteristics of tumor microenvironments.^[^ ^144^ ^]^ Conversely, external triggers are stimuli outside the body used to prompt the release or activity of chemotherapeutic agents at the tumor site.^[^ ^145^, ^146^ ^]^ Examples include ultrasound,^[^ ^147^, ^148^, ^149^ ^]^ light,^[^ ^150^, ^151^, ^152^ ^]^ heat,^[^ ^153^, ^154^, ^155^ ^]^ and magnetic field.^[^ ^156^, ^157^ ^]^ All these targeting methods can be used individually or in combination to achieve excellent targeting and effective cancer therapy (**Figure** **2**).

## 3 Current Methods for Studying and Testing Targeting and Limitations

Despite remarkable technological expansions, the drug development journey remains invariably lengthy and expensive, taking an average of 10–15 years to reach the regulatory approval stage.^[^ ^158^, ^159^ ^]^ The development of targeted cancer therapies is a multi‐stage process, beginning with developing an accurate target model, i.e., the tumor. These models are used for studying and characterization of the biochemical and physiological nature of the tumor microenvironment^[^ ^75^ ^]^ which will enable subsequent identification of unique target biomarkers. Chemotherapeutic agents targeting these biomarkers are then designed and developed, followed by efficacy and toxicity testing using biomimetic models of the target site.

Current methods include mainly the use of 2D cell culture and animal models, which are impeded by several limitations.^[^ ^58^ ^]^ These methods and their advantages and limitations are discussed in this section.

### 3.1 2D Culture

Two‐dimensional (2D) cell cultures are the most popular and widely used method for the testing of drug targeting in vitro because of their simplicity, low cost, ease of use, high viability, and high throughput.^[^ ^23^, ^24^, ^25^, ^26^ ^]^ These advantages allow the study of various factors, including drug responses, cellular mechanisms, disease pathology, and biomarker identification.^[^ ^160^ ^]^ However, 2D cultures involve alterations in cell morphology, protein expression, mitochondria content, cell polarity, cell adhesion and organization, gene expression, and cell division.^[^ ^23^, ^55^, ^56^ ^]^ These derangements subsequently impact cell signaling processes and cell biochemistry, thus inaccurately reflecting in vivo conditions.^[^ ^23^ ^]^ Moreover, the nature of 2D cultures fails to mimic the extracellular environment truly surrounding cells in vivo, hence influencing the interactions between cells and the extracellular matrix (ECM) and impacting the chemical and biological processes of cells.^[^ ^54^, ^56^ ^]^ Sedimentation is also a key limitation of 2D cultures, leading to uneven drug distribution and dosage, thus resulting in misleading findings when testing targeted agents.^[^ ^161^ ^]^ Because of these significant limitations, 2D cultures are considered poor predictors of in vivo outcomes.^[^ ^23^, ^25^, ^51^, ^56^, ^162^ ^]^

### 3.2 Animal Studies

Until recently, animal studies have been constantly required to obtain approval for clinical drug studies.^[^ ^27^, ^28^ ^]^ Although recent updates by the FDA include in vitro models as viable alternatives to animal non‐clinical tests, in vitro models have to prove capable of satisfying performance criteria.^[^ ^163^, ^164^, ^165^ ^]^ Animal studies are used to investigate drug safety and efficacy. The most common cancer animal models used for testing are xenograft rodent cancer models, such as mice and rats—immune‐compromised mice and rats in particular.^[^ ^29^, ^30^, ^31^ ^]^ Genetically engineered and chemically induced, animal tumors are also used for drug testing.^[^ ^29^, ^30^, ^31^ ^]^ Nonetheless, animal models suffer from numerous limitations that make them poor predictors of the efficacy and safety of chemotherapeutic agents in humans.^[^ ^29^, ^57^, ^58^, ^59^, ^166^, ^167^ ^]^ Discrepancies in the size, lifespan, genetic makeup, metabolic processes, and physiology between humans and animals are among the most apparent limitations faced.^[^ ^58^ ^]^ Furthermore, animal testing is highly costly and time‐consuming.^[^ ^28^, ^38^, ^39^, ^59^ ^]^ The reproducibility of animal studies is another significant concern^[^ ^40^ ^]^ and many studies found significant variability across in vivo experimentations.^[^ ^166^, ^167^ ^]^ Therefore, more accurate and reliable biomimetic models for developing and testing targeted cancer chemotherapeutics are needed.

### 3.3 Alternative Biomimetic Models

Various 3D models have recently been introduced as alternative biomimetic models to overcome the limitations of 2D cultures and animal models. These include spheroids,^[^ ^35^, ^52^, ^56^, ^168^, ^169^ ^]^ organoids,^[^ ^33^, ^40^, ^170^, ^171^, ^172^ ^]^ tissue engineering,^[^ ^41^, ^43^, ^61^ ^]^ and 3D bioprinting^[^ ^42^, ^46^, ^49^, ^60^, ^173^, ^174^, ^175^, ^176^, ^177^ ^]^ constructs. This section briefly reviews these models and discusses their advantages and limitations.

#### 3.3.1 Spheroids

Spheroids are cell cultures that grow and self‐assemble into sphere‐like structures, which promote more natural cell‐to‐cell and cell‐ECM interactions. Various methods can be used to develop spheroids^[^ ^52^, ^56^, ^168^ ^]^ using monoclonal cell cultures or co‐clonal cultures.^[^ ^56^, ^178^, ^179^, ^180^ ^]^ Tumor spheroid models have been developed to test chemotherapeutic systems equipped with different targeting modalities, including triggered targeting through magnetic waves,^[^ ^181^ ^]^ pulsed ultrasound,^[^ ^182^ ^]^ and hypoxia;^[^ ^183^ ^]^ biological targeting;^[^ ^184^, ^185^ ^]^ and others.^[^ ^34^ ^]^ Due to the 3D architecture of the spheroids, nutrients, oxygen (O_2_), metabolites, and other chemical gradients can be established and used to induce heterogeneity in the cell population with better mimicking of in vivo conditions.^[^ ^52^ ^]^ Moreover, the greater complexity and the defined geometry associated with spheroid cultures promote gene expression, cell proliferation rates, and metabolic mechanisms that differ from those of 2D cultures and better represent in vivo conditions.^[^ ^35^, ^169^ ^]^

Despite their advantages, spheroid cultures still suffer from multiple limitations, such as the lack of standard methods for testing and drug screening, challenges in growing repeatable and uniform spheroid cultures, difficulties in real‐time monitoring, and increased resistance to chemotherapy, as compared to 2D cultures.^[^ ^50^, ^52^, ^169^, ^186^, ^187^ ^]^ Furthermore, spheroids are generally synthesized under static conditions, where the mechanical forces and flow dynamics observed in vivo are absent. This can cause imbalanced perfusion rates, resulting in misleading data.^[^ ^188^ ^]^ Thus, further research is needed to improve spheroid models and overcome their limitations.

#### 3.3.2 Organoids

Organoids are derived from different stem cells cultured under conditions similar to the physiological environment to induce differentiation and self‐aggregation into clusters with organ‐like architecture.^[^ ^33^, ^40^, ^170^, ^171^, ^172^ ^]^ Organoids have been used extensively for modeling tumor microenvironments of different cancers^[^ ^189^, ^190^, ^191^, ^192^, ^193^, ^194^, ^195^, ^196^, ^197^, ^198^, ^199^, ^200^, ^201^ ^]^ and identifying potential targets that can be used for the design and development of targeted therapies.^[^ ^37^, ^189^, ^190^, ^191^ ^]^ The efficacy of targeted therapeutic agents has also been tested using tumor organoids,^[^ ^191^, ^192^ ^]^ and personalized tumor organoids have been developed using patient‐derived cells and used for targeted therapy development.^[^ ^40^, ^172^, ^193^, ^194^ ^]^ Organoids reproduce a variety of cellular interactions and biochemical processes seen in individual organs, allowing for comprehensive testing of drug targeting and efficiency.^[^ ^172^ ^]^

Despite their advantages, many challenges face the use of organoids and need to be overcome.^[^ ^52^, ^170^, ^172^, ^190^ ^]^ For example, organoid models are difficult to adapt for high throughput screening, and they have been shown to have some degree of variability.^[^ ^52^, ^172^ ^]^ Moreover, although organoids mimic numerous aspects of in vivo organ structures, vasculature, specific cell types, and significant immune and stromal factors are often absent.^[^ ^170^, ^172^ ^]^ Organoids have also been reported to reach only early stages of organ maturity, further impacting the model's accuracy in mimicking in vivo conditions and reflecting accurate drug responses and targeting efficacy.^[^ ^52^, ^170^, ^172^ ^]^ Therefore, further research and development of an accurate in vitro model for targeted drug development and testing is needed.

#### 3.3.3 Tissue Engineering and 3D Bioprinting

Tissue engineering (TE) can be used to develop constructs that are useful for modeling, which can be useful for targeted drug studies.^[^ ^41^, ^43^, ^61^ ^]^ Advances in TE can potentially be leveraged for the development of TE tumor models for evaluating targeted therapeutics.^[^ ^41^, ^43^, ^60^, ^61^, ^174^, ^195^ ^]^

However, a significant challenge with conventional TE methods is the lack of control over the positioning and distribution of cells and additives in the scaffolds, resulting in poor representation of the in vivo environment.^[^ ^60^ ^]^ To overcome this, 3D bioprinting (3DBP) has been introduced^[^ ^42^, ^46^, ^49^, ^60^, ^173^, ^174^, ^175^, ^176^, ^177^ ^]^ because it allows for controlled cell positioning and organization, which enables the development of more complex and biomimetic 3D models.^[^ ^60^, ^174^ ^]^ 3DBP tumor models have been developed,^[^ ^45^, ^48^, ^196^, ^197^, ^198^ ^]^ and they can potentially be used for studying targeted chemotherapeutics.^[^ ^199^, ^200^ ^]^ Multi‐material bioinks,^[^ ^174^, ^201^, ^202^ ^]^ some of which contain ECM components, can be combined with different cell types, biomolecules, and other additives^[^ ^60^, ^174^ ^]^ to better model tumors.

**Figure 3:**
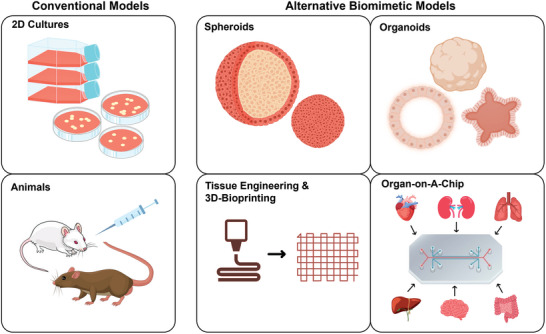
Summary of current methods for testing targeting. 2D: two‐dimensional, 3D: three‐dimensional.

Furthermore, the ability to develop personalized therapies via 3DBP constructs incorporating patient‐derived cells makes 3DBP models much more advantageous and relevant compared to animal studies.^[^ ^203^ ^]^ Unlike spheroids and organoids, vasculature can be incorporated into 3DBP models, overcoming previous limitations in nutrient balance.^[^ ^202^, ^204^ ^]^ However, despite the rapid advances in 3DBP approaches, cancer models still face significant challenges, such as difficulties in fully recapitulating the ECM, significant variability between patches, slowness, and high cost.^[^ ^61^ ^]^ Thus, further development is needed to enhance the use of 3DBP technology to produce reproducible and biomimetic tumor models that can predict in vivo outcomes with high accuracy (see **Figure** **3**).

## 4 Cancer‐On‐A‐Chip: Basics and Advantages in Studying and Testing Targeting

To address the limitations of current methods used for investigating and testing targeted chemotherapeutics,CoC systems have been developed as promising alternative biomimetic nonclinical testing platforms.^[^ ^66^, ^73^, ^75^, ^205^ ^]^ CoCs have been used to assess the safety and efficacy of targeted therapeutic agents^[^ ^73^ ^]^ and to develop personalized cancer therapies.^[^ ^74^, ^75^ ^]^ CoC models are cost‐effective, reliable, and have proven to be successful in modeling different cancers.^[^ ^66^, ^72^, ^75^, ^205^ ^]^

This section will discuss the basic concepts of CoC platforms, their advantages, and their potential use for investigating and developing targeted cancer therapeutics.

### 4.1 Basics

A deep understanding of the target cancer site's key biological, chemical, and physical features is critical for designing and developing targeted cancer therapy‐on‐a‐chip (TCToC) platforms that accurately recapitulate in vivo conditions. The development of TCToCs is based on four main aspects: chip features and components, materials and fabrication methods, cell culture, and design features.^[^ ^206^, ^207^ ^]^ These will be briefly discussed in this section.

#### 4.1.1 Common Chip Features

A variety of CoC platforms have been constructed for different cancers. CoC chip design varies depending on the cancer type; however, most CoCs share a range of common features. Early CoC models consisted of straight, parallel microchannels with an inlet and an outlet.^[^ ^206^ ^]^ Cancer mono‐ or co‐cultures are seeded in these microchannels, and culture media is introduced through the inlet to stimulate flow dynamics. These chips are often beneficial for studying early‐stage tumor development in response to different stimuli introduced into the channels, such as fibroblasts, endothelial cells, flow, chemotherapeutic agents, and others.^[^ ^206^ ^]^ However, this design restricts tissue culture interaction to lateral interaction between the microchannels, not fully representing the 3D nature of tumors.^[^ ^206^ ^]^ Later, CoCs evolved to multilayer arrangements where single‐layer CoCs are stacked on top of each other and separated by a permeable membrane, better modeling the tendency of cancerous cells to interact and grow in all directions.^[^ ^206^, ^208^ ^]^ These platforms are especially useful for cancer extravasation studies, where one layer consists of cancer cells, and the other layer(s) can model the ECM or blood/lymphatic vessels. Tumor migration and cellular response to different stimulation/treatments can also be studied.^[^ ^85^, ^96^ ^]^ Both single‐ and multi‐layer CoCs often consist of multiple microchannels, inlets and outlets, and microchambers.^[^ ^73^, ^206^ ^]^ Circular cross‐sectional designs have also been used in CoCs, depending on the application.^[^ ^209^ ^]^ Most CoCs are dynamic platforms that include fluid flow, which can be introduced using simple rocking platforms, micropumps, hydrostatic pressure, capillary forces, negative or positive pressure, or via embedded micro‐actuators, which can also be used for biophysiochemical simulation.^[^ ^67^, ^69^, ^206^, ^210^ ^]^ With the need to monitor the biophysiochemical processes and characterization, sensors and electrodes were introduced in recent CoC designs for real‐time monitoring of biochemical processes.^[^ ^90^, ^206^, ^211^ ^]^ The chip is designed to be optically transparent, allowing for optical monitoring using light, fluorescent, or confocal microscopy, and gas permeable, allowing O_2_, carbon dioxide, and sometimes water vapor diffusion. Furthermore, effluents collected from the CoCs can be analyzed to characterize pH, dissolved O_2_, genetic analysis, and biomolecules present.^[^ ^69^, ^206^, ^207^ ^]^

#### 4.1.2 Materials and Fabrication

Polydimethylsiloxane (PDMS) is the most commonly used material for the fabrication of CoCs^[^ ^206^, ^207^, ^212^ ^]^ because of its biocompatibility, inertness, transparency, permeability to gases,^[^ ^206^, ^213^ ^]^ and low cast.^[^ ^213^ ^]^ However, a significant drawback of PDMS is its hydrophobicity, resulting in the absorption of lipophilic small molecules and drugs^[^ ^212^, ^214^, ^215^ ^]^ (discussed further in the Challenges section). This limits applications in testing targeted therapeutics (e.g., pharmacokinetic /pharmacodynamic modeling studies, drug efficacy, cellular responses, drug dosages, etc.).^[^ ^216^ ^]^ Therefore, alternative materials for CoC fabrication have been explored, such as polyurethane elastomers,^[^ ^217^ ^]^ thermoplastic elastomers, polystyrene,^[^ ^218^ ^]^ acrylic‐based types of cement,^[^ ^219^ ^]^ poly(methyl methacrylate) (PMMA), and polycarbonates.^[^ ^220^ ^]^ However, thermoplastics and elastomers have demonstrated poor cell ingrowth potentials, reducing accuracy in mimicking in vivo conditions. Hydrogels or paper‐based materials may improve cell ingrowth; however, effective sterilization and leaching have become prominent challenges.^[^ ^216^ ^]^ Additionally, paper‐based CoCs have poor optical clarity, restricting the use of microscopic techniques for studying in vitro interactions in the chip.^[^ ^216^ ^]^ Therefore, the optimal material should be chosen carefully depending on the CoC design and usage. For instance, CoCs modeling shear stress and fluid flow at tumor sites should consider materials with strong mechanical properties that can handle high shear stress while mimicking biological responses, such as polyesters.^[^ ^216^, ^221^ ^]^ A comprehensive analysis of materials used for CoC fabrication can be found in ^[^ ^108^, ^206^, ^207^, ^216^, ^221^, ^222^, ^223^ ^].^

Soft lithography has been widely employed for the fabrication of CoCs^[^ ^206^, ^207^ ^]^ because it is a fast, simple, and easy technique.^[^ ^206^, ^224^ ^]^ However, for large‐scale production, soft lithography fails; instead, injection molding techniques are more often used.^[^ ^207^ ^]^ Nonetheless, injection molding techniques are associated with high initial costs related to mold fabrication.^[^ ^221^, ^222^, ^223^ ^]^ More recently, 3D printing techniques have been used for directly etching microchannels in substrates using laser micromachining, wet‐etching, or micro‐milling, 3D printing master molds to cast chips on, and resin‐ or hydrogel‐based bioprinting.^[^ ^206^, ^207^ ^]^ 3D bioprinting fabrication processes have also proven promising, including inkjet, extrusion, and laser direct technologies.^[^ ^223^ ^]^ Although relatively cheaper, inkjet and extrusion bioprinting methods risk damaging the cells by mechanical and thermal effects upon printing.^[^ ^221^, ^222^, ^223^ ^]^ While laser direct printing techniques are less damaging to cells, their high expenses are a significant limitation, in addition to limitations faced when choosing bioprinting inks.^[^ ^223^ ^]^ For comprehensive reviews of fabrication techniques, refer to^[^ ^108^, ^206^, ^207^, ^221^, ^222^, ^223^, ^225^ ^]^. Overall, fabrication techniques can be used separately or combined to overcome their limitations and produce intricate TCToCs platforms.

#### 4.1.3 Cell Culture

Initial CoC models included monotypic cultures with one cell type^[^ ^66^, ^73^ ^]^ often derived from immortalized cancer cell lines.^[^ ^194^, ^226^ ^]^ To better mimic heterogeneous in vivo environments^[^ ^212^, ^227^ ^]^ and cell interactions with other cells and ECM,^[^ ^66^ ^]^ later CoCs employed heterotypic co‐cultures with two cell types. For example, endothelial cells have been co‐cultured with breast cancer cells (MCF‐7 or MDA‐MB‐231) in a breast CoC,^[^ ^89^ ^]^ and with colon cancer cells (HCT116) in a colon cancer CoC^[^ ^227^ ^]^ to study tumor extravasation and endothelial invasion. Then, CoC cell culture evolved to include multiple cell types, including cancer‐associated fibroblasts, immune cells, epithelial cells, endothelial cells, stromal components, pericytes, and ECM elements.^[^ ^73^, ^107^ ^]^ Such heterotypic cultures can better recapitulate the complex nature of tumor microenvironments, thus predicting more accurate outcomes of targeted cancer therapeutics.^[^ ^107^ ^]^

More recent and relevant cell sources used for CoC cell cultures include induced pluripotent stem cells and primary human cells, the latter being especially useful in fabricating personalized CoCs for patient‐specific cancer therapies.^[^ ^74^, ^75^, ^76^, ^206^, ^228^ ^]^ To induce 3D, in vivo architecture, cell cultures are either directly seeded into the CoC channels using cell suspensions or cultured using the assistance of natural or synthetic matrices.^[^ ^206^ ^]^ The constructs can be cast during fabrication^[^ ^229^ ^]^ or injected^[^ ^85^ ^]^ into the channels. Collagen^[^ ^89^, ^230^ ^]^ and hydrogels^[^ ^231^ ^]^ are common matrices used to reproduce the biochemical and physical characteristics of the tumor microenvironment and/or the ECM.^[^ ^195^, ^232^ ^]^

#### 4.1.4 Designs and Applications

Single‐organ CoCs often aim to model a unique cancer cascade for an in‐depth analysis^[^ ^85^, ^233^ ^]^ or multiple mechanisms for a more comprehensive outlook^[^ ^72^, ^76^, ^85^ ^]^ of a specific cancer‐affected organ. The chip design highly depends on the type of cancer and the affected organ. For instance, lung CoC generally consists of two microchambers separated by a permeable membrane, where one chamber consists of lung cancer cells co‐cultured with airway epithelial cells and the other chamber is lined with endothelial cells to represent the alveolus.^[^ ^208^ ^]^ Similarly, glioblastoma CoC designs often consist of compartmentalized design to recapitulate the inner tumor microenvironment and outer chamber to reconstruct the ECM elements, epithelial cells, and endothelial cells.^[^ ^234^ ^]^ Other CoCs have been developed to model specific cancer cascades, including cancer growth, neovascularization, migration, and invasion.^[^ ^66^ ^]^ Vascularized systems are often designed to include scaffolds or hydrogels embedded with stromal and endothelial components.^[^ ^101^, ^233^ ^]^ CoCs studying tumor extravasation and migration similarly are often designed so that the cancer cells interface with endothelial cells in a separate chamber/channel.^[^ ^96^ ^]^

While single‐organ CoCs have been useful in mimicking in vivo conditions, they still lack organ‐organ interactions^[^ ^235^ ^]^ to investigate the spread of cancer and to study secondary and systemic drug toxicity. To overcome this, multi‐organ‐on‐a‐chip (MoC) systems have been developed^[^ ^235^ ^]^ to allow more accurate modeling of in vivo conditions.^[^ ^236^ ^]^ MoC systems have been especially useful in modeling cancer metastasis, migration, and invasion into downstream organs often called metastasis‐on‐a‐chip platforms. Up to fifteen organs have been linked in MoC platforms successfully,^[^ ^80^, ^88^, ^90^, ^99^, ^211^, ^237^, ^238^, ^239^, ^240^, ^241^, ^242^, ^243^, ^244^, ^245^, ^246^, ^247^ ^]^ and many of these models have been useful in studying the targeting efficacy and off‐target effects of targeted anticancer therapeutics.^[^ ^80^, ^88^, ^90^, ^99^, ^247^ ^]^

### 4.2 Advantages CoCs Add to Developing Targeted Cancer Therapy

There are various aspects that CoCs have that make them attractive for use in studying targeted cancer therapeutics, such as flexibility in design and precise control over microenvironment conditions, which allows for higher accuracy in replicating in vivo conditions and predicting clinical outcomes.^[^ ^64^, ^65^, ^236^ ^]^ The miniature scale of CoC devices reduces the consumption of materials, and consequently the costs.^[^ ^64^, ^65^, ^248^, ^249^ ^]^

In addition, CoC models have recently been proven to be highly valuable platforms for studying, targeting, and testing targeted cancer therapies, especially when compared to current testing methods.^[^ ^67^, ^69^, ^71^, ^80^, ^81^, ^89^, ^91^, ^96^, ^101^ ^]^ CoCs also offer new advantages over current testing methods, such as visualization and real‐time monitoring,^[^ ^67^, ^90^, ^95^, ^99^, ^100^, ^211^, ^250^ ^]^ dynamic conditions,^[^ ^67^, ^68^, ^69^, ^70^, ^71^ ^]^ and tumor microenvironment modeling.^[^ ^72^, ^81^, ^82^, ^88^, ^90^, ^208^, ^234^, ^247^, ^251^, ^252^ ^]^

#### 4.2.1 Visualization and Real‐time Monitoring

The incorporation of relevant biosensors in the chip design allows for real‐time, non‐invasive monitoring and analysis of cellular and molecular interactions,^[^ ^253^, ^254^ ^]^ mechanical forces,^[^ ^255^ ^]^ and electrical signals.^[^ ^256^, ^257^ ^]^ Employing a transparent material in CoC allows for in‐depth monitoring of cellular mechanisms and transport in each fabricated layer, which is difficult to achieve in vivo.^[^ ^96^ ^]^ Real‐time, continuous monitoring can be especially useful in identifying target molecules and understanding targeting mechanisms and efficacy, which is often difficult when using 2D culture or animal studies. Triggered chemotherapeutics, especially those utilizing internal stimuli like hypoxia^[^ ^90^ ^]^ and pH^[^ ^99^, ^100^ ^]^ have been tested using CoC models that incorporated sensors for continuous monitoring to understand their mechanism of action and targeting efficacy. To monitor hypoxia, oxygen levels were measured using integrated oxygen sensors in the CoC itself^[^ ^211^ ^]^ or by allowing an external dissolved oxygen probe access into the system through a designed ‘hole’.^[^ ^90^ ^]^ Similarly, optical pH sensors incorporated within CoC systems accurately detected pH changes in the CoC.^[^ ^100^ ^]^ Other sensors have been integrated into CoCs to monitor changes in the levels of biomarkers during cancer progression and targeted chemotherapy treatments.^[^ ^99^ ^]^ Real‐time monitoring of these biomarkers in the MoC cancer system provided key information about targeting efficacy and possible off‐target impacts of the designed targeted drug delivery system.^[^ ^99^ ^]^ Built‐in sensors achieved detailed insights into the rate of biomarker secretions in a rapid and accurate fashion, which is very difficult and time‐consuming to obtain using current testing methods.

Flow rates,^[^ ^67^ ^]^ cell invasion and migration,^[^ ^95^, ^250^ ^]^ and cell death^[^ ^100^ ^]^ have all been monitored continuously using OoC platforms integrating sensors. Real‐time flow rate tracking was achieved by tracking changes in volume using an imaging program, and time‐flow rates were easily extracted for analysis.^[^ ^67^ ^]^ Tracking flow rates is useful in understanding the transport properties of targeted chemotherapeutics.^[^ ^67^, ^258^, ^259^ ^]^ Similarly, real‐time monitoring of cell invasion was achieved non‐invasively via impedance measurements on a microfluidic device.^[^ ^250^ ^]^ While the microfluidic platform was not a fully developed OoC system, its design can be incorporated into TCToC systems to test the impact of targeted therapeutics on cell invasion and quantify targeting efficacy. Alternatively, real‐time targeted drug transport and penetration monitoring gas were achieved using non‐invasive fluorescence imaging of a breast CoC with high spatio‐temporal resolution.^[^ ^95^ ^]^ Furthermore, trans‐epithelial electrical resistance (TEER) sensors have been built into a lung CoC system, providing information regarding cytotoxicity and cell death with time during treatment with targeted chemotherapeutics.^[^ ^100^ ^]^ Hence, the ability to incorporate real‐time monitoring and visualization of processes occurring in TCToCs is a central advantage, especially in targeting studies.

#### 4.2.2 Dynamic Conditions

A key advantage of CoC systems is the ability to recapitulate dynamic in vivo conditions such as flow^[^ ^67^, ^68^, ^69^, ^70^, ^71^ ^]^ and other biomechanical cues.^[^ ^208^, ^260^, ^261^ ^]^ Flow dynamics heavily impact tumor morphology, interstitial pressure, and microenvironment; these changes all subsequently influence drug targeting and transport.^[^ ^67^, ^258^, ^259^ ^]^ Current 2D and 3D in vitro cultures are generally static models lacking continuous perfusion, thus inaccurately representing in vivo conditions as dynamic flow is absent.^[^ ^56^, ^161^, ^188^ ^]^ Meanwhile, flow stimulation can easily be integrated and controlled in TCToC platforms using a tilted rocking platform^[^ ^67^, ^70^ ^]^ peristaltic pump^[^ ^68^ ^]^ or a syringe pump.^[^ ^69^ ^]^ These systems have subsequently been used to identify potential targets^[^ ^70^ ^]^ and assess the fluid‐flow dependency of targeted therapies.^[^ ^67^, ^68^, ^69^, ^71^ ^]^ For example, elevated intratumoral pressure and rapid interstitial flow are characteristic features of pancreatic ductal adenocarcinoma (PDAC) tumors, which are difficult to reproduce in 2D and other 3D cultures.^[^ ^70^ ^]^ The PDAC TCToC platform controlled the interstitial flow, revealing a correlation between high interstitial flow and elevated multi‐drug resistant proteins (MRPs), which has been identified as a potential biomarker for targeted therapy development.^[^ ^70^ ^]^ Similarly, cerebrospinal fluid flow in choroid plexus (ChP) was successfully modeled in a leptomeningeal metastasis ChP CoC platform, where tumor morphology and enzyme profile matched in vivo conditions.^[^ ^67^ ^]^

Other CoC systems tested varying flow rates to eliminate NP sedimentation^[^ ^68^ ^]^ optimized parameters to prevent cell death via shear stress^[^ ^68^ ^]^ and investigated the relationship between fluid flow rates and drug accumulation and penetration depth.^[^ ^69^, ^71^ ^]^ Preventing sedimentation is important to accurately assess targeting efficacy because sedimentation of non‐cytotoxic NPs has been shown to cause cell death through an apoptosis‐like process.^[^ ^68^ ^]^ Moreover, while increased flow rates generally cause higher peripheral accumulation at the tumor site, studies using CoC platforms have revealed that this does not necessarily cause higher NP uptake and greater penetration depth.^[^ ^69^, ^71^ ^]^ Short interaction time between the NP and cancer cells and low bending strength can explain decreased cellular uptake at increased interstitial flow rates.^[^ ^71^ ^]^ Building on this information, targeted chemotherapeutics can be designed to increase binding strength and improve cellular uptake. Their targeting efficacy can be further tested using these biomimetic, dynamic TCToC platforms, as was done in several studies.^[^ ^67^, ^68^, ^71^ ^]^ Other biomechanical cues, such as breathing^[^ ^208^ ^]^ and heartbeating ^[^ ^260^ ^]^ have been modeled in OoCs; however, they have not yet been used to test targeting. The ability to incorporate dynamic biomechanical cues is a key advantage of TCToC systems, which can promote greater accuracy of cancer‐targeting studies.

#### 4.2.3 Advanced Tumor Microenvironment Modeling

Another advantage of CoCs is the ability to model advanced physiological and biochemical processes in the tumor at cellular and molecular levels. Various CoCs enabled the investigation of numerous molecular interactions (e.g., epithelial‐stromal crosstalk^[^ ^251^ ^]^) and genetic pathways related to cancer progression (e.g., “cell cycle checkpoint gene”^[^ ^234^ ^]^), which will be explored in further detail in the next section. Modeling tumor microenvironment accurately allowed for timely and cost‐effective target identification and targeted therapy testing.^[^ ^81^, ^82^, ^208^, ^234^, ^251^, ^252^ ^]^ Furthermore, modeling microenvironment changes during extravasation and colonization at distant organs has also been possible using MoC systems with several organs.^[^ ^72^, ^88^, ^90^, ^247^ ^]^ Similarly, these systems enabled the identification of targets that can slow down or inhibit metastasis.^[^ ^247^ ^]^ Hence, TCToCs can provide a deeper insight into the target tumor microenvironment and can potentially be used for cancer drug targeting studies.

## 5 Current Applications of Targeted Cancer Therapy‐on‐A‐Chip (TCToC)

### 5.1 Use of TCToCs to Model Target Cancer Site and Identify Potential Targets

The design of targeted chemotherapeutic agents and drug delivery systems requires an in‐depth understanding of the complex tumor microenvironment and the biochemical and physiological interactions taking place. CoCs aim to mimic the in vivo microenvironment of different cancers.^[^ ^67^, ^70^, ^79^, ^81^, ^82^, ^85^, ^86^, ^88^, ^89^, ^91^, ^93^, ^94^, ^208^, ^209^, ^227^, ^234^, ^251^, ^252^, ^262^ ^]^ Using CoC, different physiological markers have been identified as potential targets for chemotherapeutics. The following sections discuss key targets identified using CoC models, starting with the most studied cancer using CoCs.

#### 5.1.1 Breast Cancer

Breast cancer is the most common cancer in females, with ≈300 000 new cases in 2023 in the United States alone, and incidence rates continue to increase by ≈0.5% yearly.^[^ ^263^ ^]^ Breast cancer‐on‐a‐chip systems^[^ ^264^ ^]^ have been developed to model different stages of localized tumor growth^[^ ^262^ ^]^ to invasion and metastasis^[^ ^82^, ^89^, ^99^ ^]^ of ductal,^[^ ^265^ ^]^ luminal,^[^ ^251^ ^]^ and triple‐negative^[^ ^266^ ^]^ breast cancers.

Breast CoC models aided the identification of important targeting possibilities for inhibiting tumor growth and invasion.^[^ ^251^, ^262^ ^]^ For instance, the cellular architecture of ductal carcinoma was replicated in a breast CoC, and the design enabled the modeling of epithelial‐stromal crosstalk at the ECM level and quantified the transition of cells from healthy to a pathological state in real‐time;^[^ ^251^ ^]^ this was not previously possible to do except through ectopic in vivo studies. The study identified hyaluronic acid (HA), fibronectin, and collagen as key overexpressed factors during stromal activation and epithelial invasion, inducing interstitium crowding and their impact on drug transport.^[^ ^82^, ^251^ ^]^ These findings can be used to design TCTs of adequate size and shape for effective drug transport, and the breast CoC can subsequently be used for their testing. Furthermore, the identified increase in HA can serve as a potential target for new therapeutics. Similarly, breast CoC can be used to test existing HA‐targeted chemotherapies^[^ ^267^ ^]^ for non‐clinical studies.

A later breast CoC study identified PI3Kα and ErbB2 mutations as critical promoters of tumor invasion and enhanced tumor permeability, with PI3Kα mutation causing more rapid invasion.^[^ ^82^ ^]^ Accordingly, new therapeutics can be designed to target these mutations.^[^ ^268^ ^]^

However, the use of a CoC design that reproduced circulation does not fully recapitulate in vivo conditions. In addition to tumor cells, the presence of cancer‐associated fibroblasts (CAFs), endothelial cells (ECs), and immune cells were included in the system to better reproduce the tumor microenvironment.^[^ ^81^ ^]^ This advanced breast CoC model enabled the recapitulation of human epidermal growth factor 2 (HER2) overexpression in HER2‐positive breast cancers, a key target for TCT.^[^ ^81^ ^]^ Another breast CoC identified the epidermal growth factor receptor (EGFR) as a key target for anticancer treatment and inhibiting growth and migration.^[^ ^89^ ^]^

Although there have been several CoC studies on the modeling of breast cancer, only a few focused on targeting.^[^ ^81^, ^82^, ^89^, ^251^ ^]^ Other studies have successfully developed breast CoC models but have focused on studying physiological processes alone without targeting.^[^ ^209^, ^262^ ^]^ More research is needed to identify potential targets for the development of targeted chemotherapies.

#### 5.1.2 Brain Cancer

Brain cancer is one of the leading causes of cancer deaths in children and adolescents.^[^ ^263^ ^]^ However, the five‐year survival rates continue to increase over the years with advances in cancer treatment.^[^ ^269^ ^]^ Glioblastomas (GBM) are classified among the most aggressive and common brain cancers.^[^ ^94^, ^270^ ^]^ Glioblastoma‐on‐a‐chip models developed in multiple studies have proven beneficial in replicating the cancer microenvironment to identify TCT targets.^[^ ^91^, ^94^, ^234^ ^]^

Glioblastomata‐on‐a‐chip models aided the identification of critical targeting possibilities for inhibiting tumor growth and invasion. 3D GBM spheroid cultures of U87 human astrocytoma cells cultured in multi‐channel brain CoCs enabled the identification of vimentin and matrix metalloproteinases‐2 (MMP‐2) as key biomolecules for targeting tumor aggression, metastasis, and invasion.^[^ ^94^ ^]^ Continuous perfusion employed in the GBM‐chip facilitated tumor invasion studies, and this design allows for testing of already‐developed vimentin‐ and MMP‐2‐targeted chemotherapies.^[^ ^94^, ^271^, ^272^ ^]^

Although advantageous, the GBM‐chip is restricted in its ability to fully replicate in vivo brain cancer microenvironments due to the use of immortalized cancer cell lines.^[^ ^94^ ^]^ Later, improved glioblastomata‐on‐a‐chip platforms utilized patient‐derived ex‐vivo GBM spheroid cell cultures to better replicate brain cancer microenvironments.^[^ ^91^, ^234^ ^]^ Incremental hypoxia was effectively replicated in these CoCs; thus, these platforms enable testing of chemotherapeutics employing hypoxia‐triggered targeted cancer therapy.^[^ ^91^, ^234^, ^273^ ^]^ Genetic studies performed on these GBM CoCs have identified the “cell cycle checkpoint gene” as a prominent genetic factor causing tumor progression and resistance.^[^ ^234^ ^]^ Hence, this genetic mechanism can be considered a potential target for inhibiting tumor resistance. Furthermore, future studies can utilize these models to develop patient‐specific TCTs for improved cancer therapy.

In addition to GBM tumors, a brain CoC proved effective in modeling pediatric juvenile pilocytic astrocytoma using patient ex‐vivo cells cultured in an engineered microenvironment.^[^ ^67^ ^]^ While the study did not identify any potential markers for drug targeting^[^ ^67^ ^]^ the platform has great potential for TCT development and testing with further analyses.

Advances in brain CoCs continue to evolve, with glioblastoma‐on‐a‐chip models most commonly studied.^[^ ^91^, ^94^, ^234^ ^]^ Future platforms should be developed to model other brain cancers and TCT development and testing.

#### 5.1.3 Lung Cancer

Lung cancer is the primary cause of cancer incidence and mortality rates worldwide, with over 2 million cases diagnosed in 2023.^[^ ^274^, ^275^ ^]^ Approximately 80% of lung cancer deaths have been correlated with smoking, a leading risk factor.^[^ ^275^, ^276^ ^]^ CoC platforms modeling lung cancer have been developed extensively.^[^ ^93^, ^208^, ^277^ ^]^

Lung cancer‐on‐a‐chip models for TCT studies have also been developed.^[^ ^93^, ^208^ ^]^ For example, a double‐layer lung CoC system was developed to study tyrosine kinase inhibitor (TKI) therapy for non‐small‐cell lung cancer (NSCLC).^[^ ^208^ ^]^ Vacuum chambers coupled with peristaltic pumps were incorporated to recapitulate mechanical breathing and study its impact on tumor growth and therapy resistance.^[^ ^208^ ^]^ The platform revealed key insights on biomarker concentrations that can be used to develop targeted therapies. EGFR, VEGF, interleukin‐6 (IL‐6), IL‐8, and c‐MET proteins are all overexpressed in NSCLC adenocarcinomas; however, EGFR expression is downregulated, and resistance to TKI therapy increases in tumors exposed to mechanical stress caused by breathing.^[^ ^208^ ^]^ These findings can open new research paths in designing drugs targeting NSCLC tumors by considering the effects of breathing on tumor growth. In a related study, A549 spheroid cell cultures were used to construct a lung carcinoma CoC model for the evaluation of the selective cytotoxicity of a tryptophan‐rich peptide P1 against lung cancer.^[^ ^93^ ^]^ While the overexpression of the Ki‐67 biomarker was utilized to study tumor progression, it can also potentially be used as a potential target for the development of targeted cancer therapies, and their lung CoC can be used to test such targeting efficacy.^[^ ^278^ ^]^

Although many lung CoC models have shown remarkable accuracy in recapitulating in vivo environments,^[^ ^208^ ^]^ future platforms can incorporate primary cell lines to facilitate the development of targeted cancer therapies for more effective cancer treatment.

#### 5.1.4 Colorectal and Pancreatic Cancer

Colorectal cancer is the second leading cause of cancer mortality in the United States (U.S.), with incidence rates increasing by ∼9% in individuals under 55 years of age.^[^ ^279^ ^]^ Similarly, pancreatic cancer, although not as common, is among the leading causes of cancer mortality, being the third most common cause of cancer death in the U.S. and the seventh most common worldwide.^[^ ^280^, ^281^ ^]^ Due to its asymptomatic nature, early diagnosis is often difficult.^[^ ^280^ ^]^

CoC platforms modeling colorectal and pancreatic cancers have been useful in recapitulating in vivo conditions and identifying potential biomarkers for targeting.^[^ ^70^, ^227^, ^282^ ^]^

For example, the endothelial invasion mechanism of colorectal cancer was modeled in a circular 3D microfluidic CoC.^[^ ^227^ ^]^ The genetic markers Ki‐67, MMP‐1, and Caspase‐3 were effectively recapitulated and their response to treatment with gemcitabine (GEM) was studied.^[^ ^227^ ^]^ The effective CoC design shows great potential in developing and testing cancer therapies targeting genetic markers.^[^ ^278^, ^283^, ^284^ ^]^

Meanwhile, MRPs have been found to be overly expressed in PDAC CoCs due to characteristic interstitial pressure in PDACs, proving to be an important target for tackling chemoresistance.^[^ ^70^ ^]^ Interstitial pressure recapitulation was achieved using a tilted rocking platform, which simulated fluid flow.^[^ ^70^ ^]^ PDAC tumors are also characterized by different genetic mutations in humans;^[^ ^70^ ^]^ the subsequent unique phenotypes expressed could be used as markers for targeting. While PDAC CoCs cultured from human cancer cell lines are more clinically relevant, CoCs derived from genetically engineered mice proved useful in identifying and discovering potential genetic markers for TCT.^[^ ^282^ ^]^ For instance, A PDAC CoC derived from genetically engineered mice models was designed, where KPC cells containing Kras and Tris mutations and KIC cells containing Cdkn2a deletion and Kras mutation were cultured separately and in combination.^[^ ^282^ ^]^ Some overexpressed phenotypes include E‐cadherin, fibronectin, MMP‐2, and type IV collagen, all of which can be central markers for developing targeted therapies.^[^ ^271^, ^285^, ^286^, ^287^ ^]^

Thus, both genetically modified animal cancer cell lines and immobilized human cell lines have proven useful in developing colorectal and pancreatic CoC platforms for TCToC studies.^[^ ^70^, ^227^, ^282^ ^]^ However, for enhanced clinical relevance and future applications in clinical trials and precision medicine, patient‐derived cancer cells can provide more promising, applicable results when used in TCToC platforms (see **Table** **1**).

**Table 1:** Potential targets identified and modeled using cancer‐on‐a‐chip (CoC) platforms.

Cancer modeled	Cell culture	Potential targets	Reference
Breast ductal carcinoma (BDC)	MCF7 microtissues co‐cultured with normal fibroblast microtissues or cancer‐associated fibroblast (CAF) microtissues	– Platelet‐derived growth factor (PDGF) receptors – Hyaluronic acid (HA) – Spaces of fibronectin and collagen network	[251]
BDC [two mutation models: ErbB2‐amplified and PI3Kα^H1047R^]	MCF10A co‐cultured with primary human dermal micro‐ vascular cells (hMVECs)	– Human epidermal growth factor receptor (HER‐2) receptors – ErbB2 gene pathway – PI3Kα^H1047R^ gene pathway – Vascular endothelial growth factor (VEGF) receptor 2 – Interleukin‐6 (IL‐6)	[82]
HER2^+^ BDC	HER2^+^ BT474 co‐cultured with HUVEC and with or without Hs578T CAFs and peripheral blood mononuclear cells (PBMC)	– HER2 receptors	[81]
Adenocarcinoma BDC	MCF7 or MDA‐MB‐231 co‐cultured with or without HUVEC cells.	– Epidermal growh factor receptor (EGFR)	[89]
Glioblastoma	U87 human glioblastoma astrocytoma spheroids	– Vimentin – Matrix metalloproteinase‐2 (MMP‐2)	[94]
Glioblastoma	‐ U87MG human glioblastoma astrocytoma cells co‐cultured with HUVEC. ‐ Patient‐derived glioblastoma cells were cultured in GBM‐cell bioink, vascular‐cell bioink, and silicone ink, and then three dimensionally (3D) printed.	– Hypoxia (trigger) – Cell cycle checkpoint‐related gene	[234]
Glioblastoma	Patient‐derived glioblastoma tissue cultured to form spheroids.	– Hypoxia	[91]
Non‐small cell lung cancer (NSCLC), (adenocarcinoma)	Lung small airway chip: ‐ H1975 human NSCLC adenocarcinoma co‐cultured with primary human small airway epithelial cells and primary human lung microvascular endothelial cells. Lung alveolus chip: ‐ H1975 human NSCLC adenocarcinoma co‐cultured with primary human alveolar epithelial cells and human lung microvascular endothelial cells.	– EGFR – VEGF – IL‐6 – IL‐8 – Mesenchymal‐epithelial transition factor (c‐MET)	[208]
Lung Adenocarcinoma	A549 cancer cells co‐cultured with human amniotic membrane mesenchymal stem cells (hAM‐MSCs) to form 3D spheroids.	– Antigen Kiel 67 (Ki‐67)	[93]
Colorectal cancer	HCT116 colon cancer cells co‐cultured with human colonic microvascular endothelial cells (HCoMECs)	– Ki‐67 – MMP‐1 – Caspase‐3	[227]
Pancreatic ductal adenocarcinoma (PDAC)	Human S2‐028 PDAC cancer cells monoculture.	– Multi‐drug resistant proteins (MRPs)	[70]
PDAC	Two genotypes derived from genetically engineered murine pancreatic cells: KPC2 cells (with Kras and Trp53 mutations) and KIC cells (with Kras mutation and Cdkn2a deletion). The KIC cells used were of two phenotypes: epithelial (eKIC) and mesenchymal (mKIC). Five culture conditions were applied: monocultures of KPC2, eKIC, and mKIC, KPC2 co‐cultured with mKIC, and mKIC co‐cultured with eKIC.	– E‐cadherin – MMP‐9 – Fibronectin – Type IV collagen	[282]
B‐cell acute lymphoblastic leukemia (B‐ALL)	Three main culture conditions were employed: B‐ALL cells, niche cells, and B‐ALL cells co‐cultured with niche cells. Niche cells consisted of vascular endothelial (ECs), perivascular mesenchymal stem cells (MSCs), and endosteal osteoblasts. ‐ B‐ALL cells of different genotypes were used, including murine (Ph+ GFP+), human (EVT6‐RUNX1 REH, MLL RS(4;11), E2A‐PBX1 697, E2A‐HLF UOCB1, and NALM‐6, Ph+ SUP‐B15) and patient‐derived (Ph+ B‐ALL blasts and non‐Ph+ B‐ALL blasts) B‐ALL cells. ‐ Murine (C166) and human (HUVEC) epithelial cells ‐ Murine MSCs (OP9) and human BM stem cells hMSCs, cord blood cells (CD34+ cells), and BM mononuclear cells. ‐ Human osteoblast cells (hFOB 1.19).	– Chemokine ligand 5 (CCL5) – CCL2 – IL‐6 – IL‐8 – Ki‐67 – Nuclear factor kappa‐light‐chain‐enhancer of activated B cells (NF‐κB) pathway – Stromal cell‐derived factor‐1 (CXCL12) and chemokine (C‐X‐C motif) receptor 4 (CXCR4) – Vascular cell adhesion molecule‐1 (VCAM‐1) /Very late antigen‐4 (VLA‐4)	[252]
Ovarian endometroid adenocarcinoma	A2870 epithelial ovarian cancer cells co‐cultured with human ovarian microvascular endothelial cells (HOMECs)	– Glycoprotein VI (GPVI) – galectin 3	[85]

#### 5.1.5 Leukemia

Leukemia is the leading childhood cancer worldwide and is also highly common in adolescents.^[^ ^263^ ^]^ Furthermore, leukemia is the second‐leading cause of death in children in the United States.^[^ ^263^ ^]^ A leukemia‐on‐a‐chip model was developed, and an extensive molecular analysis of the different niches in B‐cell acute lymphoblastic leukemia (B‐ALL) was carried out.^[^ ^252^ ^]^ The circular channel‐in‐channel design recapitulated the bone's medullary cavity and central sinus, effectively mimicking the in vivo leukemia microenvironment and biomolecule expression.^[^ ^252^ ^]^ Findings revealed multiple pathways and biomolecules that serve as potential therapeutic targets in the REH and SUP B‐ALL subtypes, such as CCL5, CCL2, IL‐6, IL‐8, and Ki‐67. Furthermore, key potential target signaling pathways identified include the NF‐κB pathway, which is affected by CXCL12/CXCR4 and VCAM‐1/VLA‐4 pathways.^[^ ^252^ ^]^ Hence, leukemia‐on‐a‐chip platforms show great potential for applications in testing targeted chemotherapeutics.

#### 5.1.6 Ovarian Cancer

Ovarian cancer is the fifth leading cause of cancer mortality in women, with higher incidence rates in older women.^[^ ^288^, ^289^ ^]^ However, incidence and mortality rates have declined over the years.^[^ ^288^, ^289^ ^]^ A CoC platform modeling the ovarian cancer tumor microenvironment, focusing on platelet‐cancer cell interaction—due to their importance in tumor invasion and metastasis—has been developed.^[^ ^85^ ^]^ A 3D organotypic chip was achieved with a multilayer, multichannel design: an upper tumor culture chamber and a lower vascular chamber separated by a membrane; platelet extravasation from the lower vascular chamber to the upper tumor chamber was monitored, and platelet's role in triggering cancer cell migration into the side ECM chambers was recapitulated, facilitating in‐depth study of tumor microenvironment and mechanisms for TCT.^[^ ^85^ ^]^ The binding of glycoprotein VI (GPVI) molecules in platelets with galectin‐3 in cancer cells was identified as a key interaction that promotes metastasis. Both GPVI and galectin‐3 can be targeted to inhibit tumor invasion.^[^ ^85^ ^]^ Hence, the platform can be used to design and test cancer therapies targeting GPVI and galectin‐3. The platform could be improved by incorporating immune factors and induced pluripotent stem cells (iPSCs) for a more comprehensive and personalized treatment development.^[^ ^85^ ^]^ Furthermore, ovarian CoC platforms studying targeted cancer therapies are minimal; new platforms are needed to better study ovarian cancer and develop TCTs.

While many identified target biomarkers were discovered earlier without the need for CoC platforms, successful modeling of these markers on a chip in vitro to mimic in vivo conditions is especially critical because it facilitates targeting studies and rapid development of TCTs (**Table** **2**).

**Table 2:** Potential targets identified and modelled in single‐ and multi‐organ metastasis‐on‐a‐chip platforms.

Cancer	Tumor microenvironment	Metastasis step	Cell culture	Potential target	Reference
Glioblastoma (GBM)	GBM tumor	Angiogenesis	GL261 or CT‐2A GBM cancer cells co‐cultured with endothelial cells (C166‐GFP) and macrophages (RAW264.7)	– Transforming growth factor‐β (TGF‐β) – TGFβ Receptor 1(TGFβ‐1) – Interleukin‐10 (IL‐10) – Integrin αvβ3	[86]
Nasopharyngeal carcinoma (NPC)	NPC tumor	Invasion	NPC‐BM1 cells	– IL‐6	[250]
Human mammary adenocarcinoma (hMAC)	Bone	Extravasation	MDA‐MB‐231 cells co‐cultured with human bone marrow‐derived mesenchymal stem cells (hBM‐MSCs) and human umbilical vein endothelial cells (HUVECs)	– chemokine (C‐X‐C motif) receptor 2 (CXCR2) – CXC ligand 5 (CXCL5)	[87]
hMAC	Bone	Extravasation	MDA‐MB‐231 cells co‐cultured with hBM‐MSCs, osteoblast‐differentiated primary hBM‐MSCs, and primary HUVECs	– Adenosine – A3 adenosine receptor (A3AR)	[233]
Breast cancer leptomeningeal metastasis	Human brain choroid plexus (ChP)	Colonization	MCF‐7 or SKBR3 cancer cells co‐cultured with primary human brain microvascular endothelial cells (hBMEC), human brain vascular pericytes (hBVP), and human choroid plexus epithelial cells (hCPEPiC)	– Human epidermal growth factor 2 (HER2) – Cluster of differentiation 47 (CD47)	[67]
Colon carcinoma	Multi‐organ CoC with two chambers: Colon cancer and liver chambers	Migration to healthy liver	Colon cancer chamber: HCT‐116 or SW480 co‐cultured with human intestine epithelial cells INT‐407 Liver chamber: HepG2 cells	– N‐cadherin – Proliferating cell nuclear antigen (PCNA) – Marix metalloproteinases (MMPs) – B‐catenin – Zonula occludens ((ZO)‐1) – Vinculin	[88]
Non‐small cell lung cancer (NSCLC)	Multi‐organ CoC with two chambers: lung cancer and healthy liver chambers	Metastasis to liver	Lung cancer chamber: A549 cells co‐cultured with HFL‐1 fibroblasts. Liver chamber: L02 liver cells	– Hypoxia inducible factor 1 alpha (HIF‐1α) – Snail 1 – Snail 2 – TGF‐β1 – Wingless‐related integration site (Wnt) – Nuclear factor kappa‐light‐chain‐enhancer of activated B cells (NF‐κB) pathway – Claudins – MMPs – Vimentin – Alpha fetoprotein (AFP) – Gamma‐glutamyl transpeptidase (γ‐GT) – Alpkaline phosphatase (ALP)	[90]
NSCLC	Multi‐organ CoC with four chambers: Lung cancer, brain, bone, and liver chambers	Migration to and colonization of the brain, bone, and liver	Lung cancer: A549 lung cancer cells co‐cultured with HUVECs, fibroblasts (W138), bronchial epithelial cells (16HBE) and monocytes (THP‐1) Brain: astrocytes (HA‐1800) Bone: osteoblasts (Fob1.19) Liver: hepatocytes (L02)	– AFP – receptor activator of nuclear factor kappa beta (RANKL) – CXCR4	[247]

#### 5.1.7 Metastasis

Metastasis occurs in a plentiful of cancers, including prostate, breast, and lung cancer, among others.^[^ ^290^, ^291^ ^]^ Metastatic cancers represent a growing burden, as it is responsible for over two‐thirds of cancer deaths.^[^ ^290^, ^291^, ^292^ ^]^

In addition to CoC models studying local tumor environments, many studies have developed specific CoC and MoC models to study cancer metastasis, often called “metastasis‐on‐a‐chip”.^[^ ^72^ ^]^ Metastasis involves four critical stages, angiogenesis, the formation of new vessels; intravasation, where metastatic cancer cells interact with the endothelial barrier; extravasation, where cancer cells escape and circulate in the blood; and, finally, colonization at a new tissue site.^[^ ^72^ ^]^ Single CoC and MoC models for the different metastatic stages have been developed to better understand the underlying mechanisms of cancer metastasis, and they have been extensively reviewed.^[^ ^72^ ^]^

So far, metastasis‐on‐a‐chip platforms have provided useful insight into tumor mechanisms, which can be used to identify potential targets for chemotherapy and test TCTs. These platforms, including angiogenesis/vasculature‐on‐a‐chip platforms, will be discussed below.

##### Single Organ Metastasis‐on‐A‐Chip

Angiogenesis/vasculature‐on‐a‐chip platforms for different cancers have helped identify new biomarkers for targeted therapies inhibiting metastasis. For example, a glioblastoma‐angiogenesis CoC model was developed to investigate angiogenesis‐related mechanisms as well as macrophage‐associated immunosuppression.^[^ ^86^ ^]^ The impact of different macrophage phenotypes was effectively modeled, where M2 macrophages were found to promote angiogenesis in glioma tumors depending on the subsequent secretion of cytokines. Important cytokines identified include transforming growth factor beta (TGF‐β) and IL‐10, which serve as potential targets that can be used to achieve anti‐angiogenesis. Alpha‐v beta‐3 (α_v_β_3_) integrin and TGFβ receptor type 1 (TGFβ‐1) were also identified as key targets for anti‐angiogenesis therapy.^[^ ^86^ ^]^ While the angiogenesis CoC model provided valuable insight into important targets, murine‐based glioma organoid cultures were used, thus limiting the clinical relevance of the model. Vasculature‐on‐a‐chip models have also been used to test targeting and targeted drug delivery, which will be discussed in more detail in sections 5.2 and 5.3.^[^ ^80^, ^83^, ^96^ ^]^

Similarly, intravasation and cell invasion were modeled in single‐organ CoC platforms that facilitated the identification of therapeutic targets. For example, using a nasopharyngeal carcinoma‐on‐a‐chip, the cytokine IL‐6 was identified as a key ECM component promoting cancer cell invasion and metastasis.^[^ ^250^ ^]^ This can be exploited as a target to inhibit invasion and metastasis (intravasation in particular, which IL‐6 promotes). The nasopharyngeal carcinoma‐on‐a‐chip was unique in its design, employing electrodes for real‐time measurements of impedance, which facilitated real‐time monitoring of cell invasion and quantitative analysis of IL‐6's impact on intravasation.^[^ ^250^ ^]^

Moreover, colonization of metastasized breast cancer cells to the bone^[^ ^87^, ^233^, ^293^ ^]^ and brain^[^ ^67^ ^]^ has been effectively modeled in multiple studies using single‐organ CoCs. A bone‐on‐a‐chip model effectively recapitulated in vivo interactions between osteoblastic tissues and breast cancer cells using a unique murine‐human co‐culture, capturing the early physiological mechanisms of metastasis and colonization of breast cancer cells in the bone.^[^ ^293^ ^]^ The model's clinical relevance is reduced due to the use of murine cultured and the study did not focus on identifying potential therapeutic targets; nonetheless, the bone OoC proved very promising for future targeting studies.

Furthermore, extravasation and specificity of breast cancer cells, MDA‐MB‐231, was modeled in bone‐microenvironment OoC platforms.^[^ ^87^, ^233^ ^]^ The CXCR2 surface cell receptor was found to play a pivotal role in promoting extravasation.^[^ ^87^ ^]^ In contrast, adenosine and its receptor A_3_AR were key inhibitors of extravasation and cancer metastasis,^[^ ^233^ ^]^ thus both proving to be essential biomolecules for TCT. Similarly, the chemokine CXCL5 secreted by bone cells was identified as a target molecule and an important factor promoting the extravasation of breast cancer cells to bone microenvironments due to its interaction with CXCR2 receptors in breast cancer cells.^[^ ^87^ ^]^ Meanwhile, breast cancer leptomeningeal metastasis to the brain on a human choroid plexus‐on‐a‐chip platform was developed, mimicking in vivo cerebral spinal fluid flow dynamics and recapitulating the high levels of HER2; this is similar to what is seen in HER2+ breast cancer.^[^ ^67^ ^]^ Furthermore, CD47 expression was upregulated, indicating that this can also be a potential target for therapy.^[^ ^67^ ^]^ In addition to identifying potential targets, developed OoC was promising for testing targeted therapies, which will be discussed further in section 5.2.

##### Multi‐Organ Metastasis‐on‐A‐Chip

In addition to single OoC models for studying metastasis, several multi‐organ CoCs have been developed to recapitulate metastasis from the cancer site to different organs. These platforms can be used to better understand organ‐organ interactions and metastatic mechanisms, allowing for the identification of critical biomolecules that can be utilized for targeting. For example, a two‐organ MoC platform modeled the gut and liver in two separate chambers, interconnected by vessel‐like channels, to recapitulate colorectal cancer metastasis to liver tissues.^[^ ^88^ ^]^ N‐cadherin and proliferating cell nuclear antigen (PCNA) were overexpressed in both gut and liver tissues, indicating metastasis of colorectal cancer cells and colonization at the liver site. Gut cancer cells further had overexpressed MMPs, all of which can be used as targets. B‐catenin, ZO‐1, and vinculin were identified in liver and gut microenvironments, but at lower concentrations compared to PCNA and N‐cadherin; nonetheless, they can serve as potential targets for therapy.^[^ ^88^ ^]^ Moreover, lung cancer metastasis to the liver was investigated via a lung‐liver MoC platform, which enabled the control of O_2_ levels and the study of hypoxia by passing O_2_ gas of different concentrations through micropipes.^[^ ^90^ ^]^ Dissolved O_2_ measurements were incorporated into the chip using an O2 probe.^[^ ^90^ ^]^ A key target identified was the hypoxia‐inducible factor 1 alpha (HIF‐1α), which played a significant role in promoting epithelial‐mesenchymal transition (EMT) and activating downstream factors, including Snail 1 and Snail 2, thus enhancing metastasis.^[^ ^90^ ^]^ Other EMT markers detected at high levels included TGF‐β1, Wnt, NF‐κB, claudins, MMPs, and vimentin.^[^ ^69^ ^]^ Moreover, alpha‐fetoprotein (AFP) was expressed at high levels in liver cells, indicating colonization of metastatic lung cancer cells in the liver. AFP can be used to target metastatic cancer in the liver, in addition to gamma‐glutamyl transpeptidase (γ‐GT) and alkaline phosphatase (ALP).^[^ ^69^ ^]^ Later studies expanded upon this to develop a 4‐organ MoC platform investigating lung cancer metastasis to the brain, bone, and liver.^[^ ^247^ ^]^ The platform effectively recapitulated cancer cell migration and colonization at distant organs, as demonstrated by the over‐expression of AFP, RANKL, and CXCR4 protein in liver, bone, and brain cells. These biomolecules can potentially be used to develop and investigate targeted anti‐metastasis therapies.^[^ ^247^ ^]^ Thus, MoC cancer platforms proved useful for studying cancer metastasis and can equally be useful for developing and investigating TCT.

**Figure 4:**
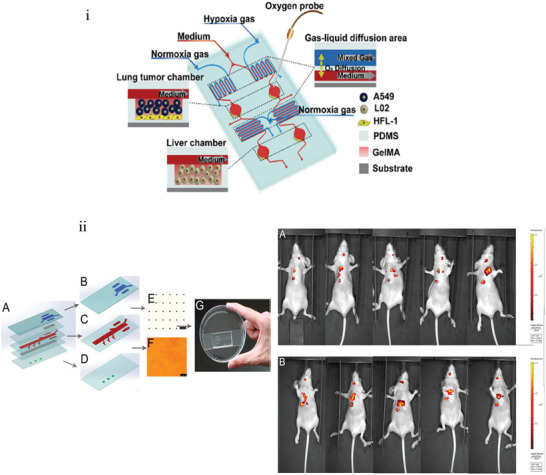
Single‐ and multi‐organ metastasis‐on‐a‐chip platforms i) Schematic diagram of 3D‐CMOM system with description of the function of each area on the chip. Reproduced with permission.^[^ ^90^ ^]^ Copyright 2021, ACS Publications. ii) Design of a multi‐organs‐on‐a‐chip to mimic lung cancer metastasis and in vivo validation of system performance. Reproduced with permission.^[^ ^247^ ^]^ Copyright 2016, ACS Publications.

All the above‐mentioned models listed in Table 2 have potential for use in more detailed analyses of the in vivo mechanisms and cellular processes in an easy, more accessible in vitro device; thus, findings from these CoCs and metastasis‐on‐a‐chip models can be used to identify potential targets for the design of targeted therapeutic agents (**Figure** **4**).

#### 5.1.8 Vascularization

The formation of blood vessel networks at tumor sites is critical for tumor growth and cancer metastasis.^[^ ^294^ ^]^ Angiogenesis, the formation of new blood vessels, at the tumor site, facilitates adequate supplies of nutrients and oxygen to cancer cells and waste removal, in turn leading to cancer progression and growth.^[^ ^294^ ^]^ CoCs modeling vascularization and angiogenesis at target cancer sites can provide more information on the mechanisms involved, enabling the identification of target biomolecules and pathways for TCT development. However, the dynamic nature of vascularization and blood flow in these regions is complex to mimic. Careful consideration of flow dynamics, cell structure and growth, mechanical stresses, and nutrients provided is needed to develop a biomimetic vascularization‐on‐a‐chip platform for TCT development and testing.^[^ ^86^, ^96^, ^111^, ^294^, ^295^ ^]^ Cellular components required for accurate recapitulation on *in* vivo vascular architecture at tumor sites include endothelial cells, pericytes, and fibroblasts, combined with extracellular components like integrins, MMPs, fibrinogens, collagens, and others.^[^ ^295^, ^296^, ^297^ ^]^ Meanwhile, mechanical factors include oxygen gradients, sheer stress, interstitial pressure, and others.^[^ ^294^, ^295^, ^296^, ^297^, ^298^ ^]^ The research focus defines which factors are included in the CoC design and which can be excluded, as including all factors increases complexity and costs.

For applications in TCT development and testing, several vasculature‐on‐a‐chip platforms of different cancer types have been explored.^[^ ^80^, ^83^, ^85^, ^86^, ^96^, ^209^ ^]^ Angiogenic sprouting in glioblastoma models was recapitulated by seeding endothelial cells in a collagen hydrogel channel, with inversion at different time points for cell growth along the lumen circumference.^[^ ^86^ ^]^ This design effectively resembled vasculature at the tumor site and was situated in parallel to GBM cancer cell channel in a CoC platform.^[^ ^86^ ^]^ Combined with macrophages and cytokines infusion, integrin αvβ3 and TGFβ‐R1were identified as key contributors to angiogenesis; thus, the vascularized GBM CoC platform facilitated the identification of biomarkers for TCT development. However, the use of murine cell cultures limits the clinical translation of this model.^[^ ^86^ ^]^ Collagen hydrogel‐based channels also proved effective in modeling orthotopic lung cancer vasculature, adopting a similar method of endothelium cell culturing along lumen circumference.^[^ ^208^ ^]^ In addition to identifying biomarkers supporting angiogenesis (EGFR, VEGF, and cytokines (IL‐6, IL‐8)), the impact of mechanical breathing stresses on vascularization and expansion was studied, using a vacuum pump. Meanwhile, Saha et al. ^[^ ^85^ ^]^ investigated the mechanical impact of vascularization shear in ovarian CoC platforms and identified GPVI and galectin to be most affected. Moreover, platelets were co‐cultured with the cancer cells, to study platelet‐cancer interactions underflow dynamics in vitro.^[^ ^85^ ^]^ Hence, biomarkers and mechanical stresses have been effectively modeled in vascularized CoCs, with great potential for use in TCT development and testing.

Other studies investigate the impact of varying genetic factors on vascularization and cancer progression through vasculature‐on‐a‐chip cancer platforms. Kutys et al. ^[^ ^82^ ^]^ assembled a 3D extracellular matrix with an embedded perfusable endothelium, in adjacence to a channel with ductal epithelium and fluid‐filled lumen, to recapitulate vascularization in breast cancer. Mutations in different genetic pathways were introduced, and the morphogenic phenotype changes in vasculature and biomarker expression were visualized using the CoC.^[^ ^82^ ^]^ Future uses for testing targeted chemotherapeutics can further add value to the biomimetic model, especially TCTs targeting genetic pathways.

Fibrinogen hydrogels embedded with ECM factors demonstrated great accuracy in mimicking vascularization of breast cancer metastasis to the bone marrow.^[^ ^233^ ^]^ More recently, Agarwal et al. ^[^ ^101^ ^]^ introduced an advanced vascularized CoC platform, whereby microtumors are first assembled in a core‐shell structure using a type‐1 collagen core and an alginate hydrogel shell. The microtumors are then aggregated into a 3D matrix block with stromal and endothelial cells in a collagen‐based hydrogel with dynamic perfusion. The intricate recapitulation of vasculature within a CoC platform, although not contributing to biomarker identification, facilitated studying targeted drug delivery and its impact on vascularization and tumor growth, contributing to the development of TCTs.^[^ ^101^ ^]^

**Figure 5:**
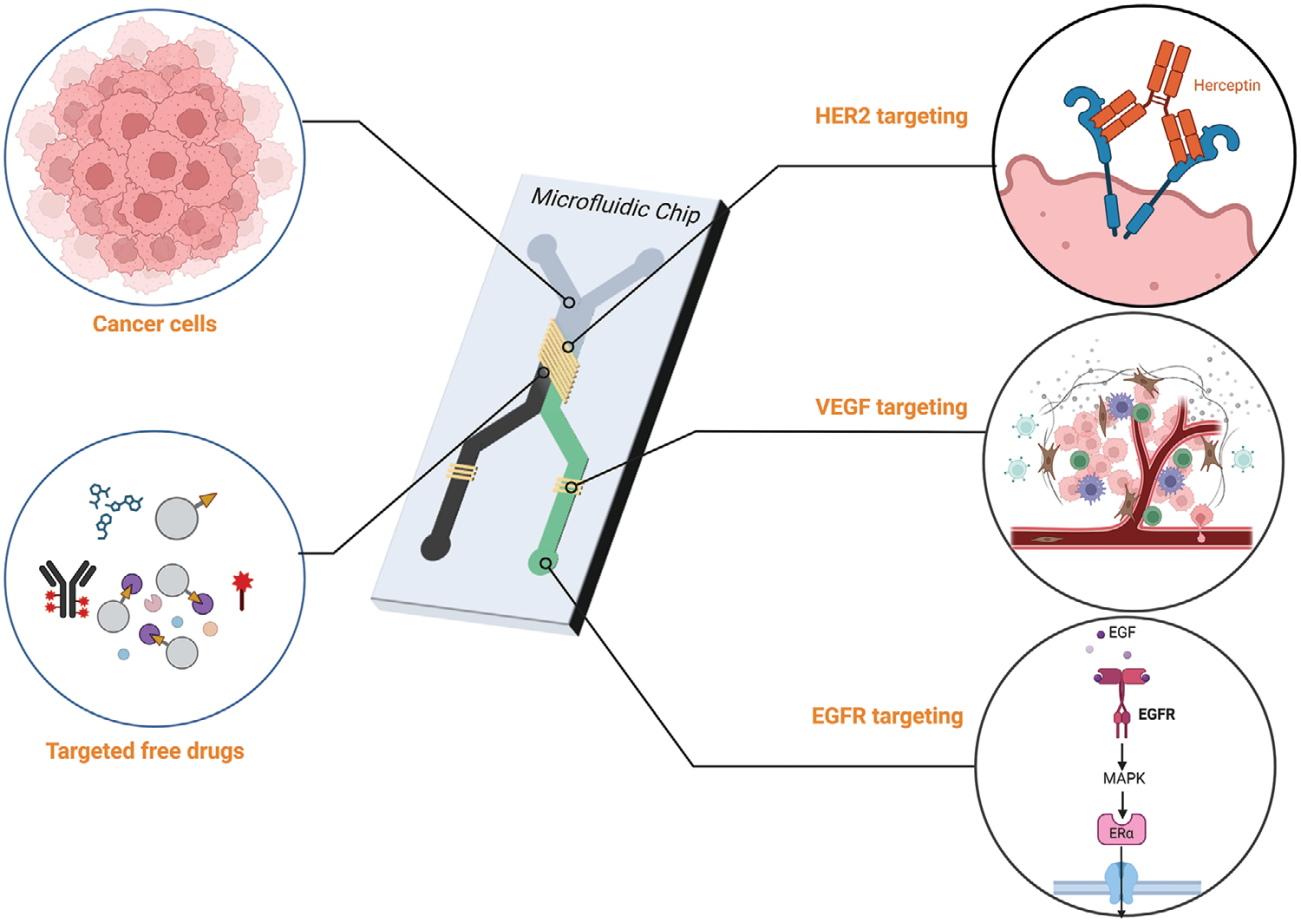
Evaluating targeted chemotherapeutics in CoC models (Created using BioRender.com). HER2: Human epidermal growth factor receptor 2, VEGF: Vascular endothelial growth factor, EGFR: Epidermal growth factor receptor.

Overall, integrating dynamic vasculature in CoC platforms has proven effective and accurate in recapitulating in vivo tumor microenvironments. Using these vascularized models can expedite TCT development and testing, thus minimizing time to clinical translation. However, the short life time and functionality of vascularized models are key limitations.^[^ ^296^ ^]^ Moreover, the micro‐scale nature of vascularization at tumor sites requires sophisticated techniques for accurate recapitulation. Bioprinting shows great promise for overcoming this limitation, although it introduces challenges related to bioinks used and materials.^[^ ^297^ ^]^ Integration of spheroids and organoids into vasculature‐on‐a‐chip models also shows great promise^[^ ^298^, ^299^ ^]^ and future research should investigate exploring the applications of vascularized CoCs in TCT development and testing (see **Figure** **5**).

#### 5.1.9 Epithelial‐to‐Mesenchymal Transition

Epithelial‐to‐mesenchymal transition (EMT) involves the transition of epithelial cells into mesenchymal phenotypes, losing cell junctions and epithelial markers.^[^ ^300^, ^301^ ^]^ In cancer, EMT is incomplete, producing abnormal tumor cells with hybrid epithelial and mesenchymal phenotypes that tend to aggregate and cluster.^[^ ^300^ ^]^ EMT is widely recognized as a dynamic, continuous driving force in cancer metastasis and progression.^[^ ^300^, ^302^ ^]^ Cancer cells are stuck in the transition phase with both epithelial and mesenchymal properties, exhibiting improved survival, therapy resistance, and metastasis.^[^ ^302^ ^]^ Several factors contribute to EMT progression in cancers, including cytokines, hypoxic conditions, secreted growth factors, metabolic changes, stromal crosstalk, chemotherapeutic treatments, and other translational factors.^[^ ^300^, ^301^, ^302^ ^]^ Understanding the molecular mechanisms of EMT at cancer sites can uncover critical biomarkers and pathways for TCT development.

CoC platforms have demonstrated great efficacy in recapitulating EMT in vitro, enabling closer study of related mechanisms and identification of target biomarkers.^[^ ^89^, ^90^, ^303^, ^304^, ^305^, ^306^ ^]^ For instance, a multi‐organ lung cancer–liver metastasis CoC platform facilitated studying EMT‐driven metastasis under hypoxic conditions.^[^ ^90^ ^]^ Careful control and monitoring of oxygen levels, using oxygen sensors and gas channels, allow for effective recapitulation of the hypoxia‐inducible factor 1α (HIF‐1α) pathway. Subsequently, EMT activation and cancer metastasis to liver chambers were illustrated through Snail 1 and 2 pathways. The accurate replication of EMT pathways in vitro using this CoC platform has great potential for testing and developing TCT.^[^ ^90^ ^]^

Other studies adapted single‐organ CoC to replicate EMT activation in lung cancers.^[^ ^303^, ^304^, ^306^ ^]^ Guo et al. ^[^ ^306^ ^]^ co‐cultured NSCLC A549 cancer cells with NCI‐HI975 macrophages in adjacent, parallel chambers, to model stroma crosstalk and its impact on EMT. The CoC platform effectively modeled E‐cadherin under expression and N‐cadherin and Slug overexpression. It also revealed EMT activation upon inducing αB‐Crystallin (CRYAB) overexpression.^[^ ^306^ ^]^ Similarly, a multichannel, 3D NSCLC CoC identified vimentin overexpression as a key indicator of EMT activation and cancer metastasis; EMT was further enhanced under dynamic flow shear stresses generally observed in vivo and successfully recapitulated in the lung cancer OoC.^[^ ^304^ ^]^ Meanwhile, Aref et al. ^[^ ^303^ ^]^ adopted a different design, whereby lung adenocarcinoma tumor spheroids were embedded in a 3D hydrogel‐based scaffold, in proximity to endothelial monolayer cultures. The CoC platform proved highly effective in TCT testing of multiple EMT‐targeted chemotherapies, illustrating accurate recapitulation of tumor microenvironments.^[^ ^303^ ^]^ While used for TCT testing, the platform can further be studied to identify new EMT activation and inhibition pathways and design new targeted therapies.

Furthermore, recapitulation of EMT mechanisms in breast cancers‐on‐a‐chip has great promise for biomarker identification and TCT development.^[^ ^89^, ^305^ ^]^ A double‐channel lymph vessel–blood vessel biomimetic breast CoC platform replicated EMT activation and angiogenesis of breast cancer in lymph and blood vessels.^[^ ^305^ ^]^ Using CoC platform, inflammatory cytokines (IL‐6) were introduced, illustrating their key role in driving EMT and subsequent metastasis initiation; this highlights IL‐6 as an important target for TCT development.^[^ ^305^ ^]^ Meanwhile, Azadi et al. illustrated EMT responses to EGFR‐targeted chemotherapy, where treatment inhibited EMT‐driven cancer invasion by reducing vimentin levels and increasing E‐cadherin.^[^ ^89^ ^]^

Overall, CoC platforms have demonstrated great promise for modeling EMT mechanisms and subsequent cancer metastasis. Different key biomolecule targets have been highlighted through these platforms, and their use for TCT development is a promising future direction.

### 5.2 Testing Targeted, Non‐encapsulated (Free) Chemotherapeutics

In addition to identifying several potential targets, CoCs have been especially useful as in vitro models for testing targeted chemotherapeutic agents and predicting in vivo results. In fact, many of the previously discussed OoC studies have demonstrated the efficacy of targeted drugs in inhibiting tumor growth and reducing metastasis through single‐ and multi‐organ CoC platforms. Free chemotherapeutic drugs utilizing either active or triggered targeting methods have been tested and will be discussed in the following sections.

#### 5.2.1 Testing Active Targeting Drugs

##### HER2 Targeting

OoC has been used to test various agents that target HER2. For instance, the efficiency of Trastuzumab, which targets HER2 receptors in breast cancer, has been investigated using CoC to determine its targeting efficiency.^[^ ^67^ ^]^ It was found that the SKBR3 cell line, which overexpresses HER2, had higher cell death rates as compared to the MCF‐7 cells, which lack HER2 receptors, indicating effective targeting. In addition, trastuzumab had no significant impact on SKBR3 in static cultures, highlighting the role of dynamic conditions.^[^ ^67^ ^]^ In another breast CoC study, Trastuzumab targeting efficacy was also demonstrated, showing an effect on BT474 cells (HER2‐ overexpressing) and no significant effect on MCF‐7 cells.^[^ ^81^ ^]^ Results obtained from the TCToC studies are comparable to published clinical results, which have proven trastuzumab's efficacy in targeted breast cancer treatment.^[^ ^307^, ^308^ ^]^ However, late‐phase clinical studies discovered cardiotoxic effects of Trastuzumab, indicating weak targeting ability;^[^ ^309^ ^]^ this was not discovered in CoC platforms due to the limited study of single organs. The development of multi‐organ CoCs can help discover such off‐target effects and toxicities, thus improving the accuracy and clinical relevance of CoCs for testing TCTs. Nonetheless, the promising results above are a step towards using CoCs as preclinical platforms for testing targeted chemotherapeutics.

##### VEGF Targeting

CoC platforms were used to study VEGF targeting. For example, a mammary duct CoC used for studying the chemotherapeutic agent inhibiting VEGFR2, Semaxanib, showed that Semaxanib leads to the suppression of VEGF expression in breast cancer cells.^[^ ^82^ ^]^ Compared to published results in animals, Semaxinab showed similar efficacy in inhibiting VEGF‐2 receptors and subsequently inhibiting breast cancer metastasis.^[^ ^310^, ^311^ ^]^ Initial clinical trials have proven Semaxinab's efficacy in inhibiting VEGF expression; however, the drug was later withdrawn after clinical trials due to its off‐target toxicity.^[^ ^311^ ^]^ Sorafenib is another agent that targets both VEGFR and PDGF receptor‐β and inhibits Raf, a serine kinase expressed in colorectal carcinomas.^[^ ^83^ ^]^ Treating angiogenesis‐on‐a‐chip models with Sorafenib led to the regression of new vasculature formations and reduced vessel lengths.^[^ ^83^ ^]^ The use of vascularized‐ colorectal ToC investigating concentration‐ and time‐based response showed that doses of 1 µM were inactive, and the IC_50_ was defined as 21 µM.^[^ ^84^ ^]^ However, Sorafenib's potency against cancer cells on a chip was four times greater than that against epithelial cells, indicating efficient targeting. CoC is thus possible to use for performing drug safety tests and dose‐response analyses.^[^ ^84^ ^]^ Moreover, compounds targeting both VEGFR2 and PGDF receptors, such as pazopanib and axitinib, had higher anti‐angiogenesis efficacy as compared to compounds exclusively targeting VEGFR, such as vandetanib and apatinib.^[^ ^83^ ^]^ These results agree with clinical studies, as apatinib has been approved for targeted treatment of solid cancers in China and is a candidate in ongoing phase II/III clinical trials for treating other cancers.^[^ ^312^, ^313^ ^]^ It was found that the most effective anti‐angiogenesis targeting agents were those targeting VEGFR, PGDFR, and Tie2, as demonstrated by treating vasculature chips with cabozantinib and linifanib.^[^ ^83^ ^]^ Both cabozantinib and linifanib have also shown promising anti‐angiogenesis results in vivo and in clinical studies.^[^ ^314^, ^315^, ^316^ ^]^ The investigation of several targeted drugs easily and rapidly via these OoC platforms proves that OoCs are highly effective in vitro models for studying targeting. Furthermore, study findings highlight that CoCs are promising preclinical platforms for in vitro testing of TCTs, due to their high agreement with in vivo and clinical study results.

##### GPVI Targeting

An ovarian cancer‐on‐a‐chip was used to test the targeting efficacy of Revacept, an anti‐GPVI monoclonal antibody, and its impact on platelets in the tumor.^[^ ^85^ ^]^ The effect of targeting with Revacept, which targets GPVI in inhibiting proliferation and suppressing the invasions of ovarian cancer, was demonstrated using ovarian CoC that employed A2870 and OVCAR3 cancer cell lines.^[^ ^85^ ^]^ These results agree with in vitro results found when treating colon cancer with Revacept.^[^ ^317^ ^]^ However, Revacept has yet to be tested in vivo on animal models. Results from the CoC need to be validated with future animal studies and clinical trials to understand their potential as pre‐clinical testing platforms.

##### Dual TGFB‐R1 and Integrin αvβ3 Targeting

The use of CoC was useful in demonstrating the effectiveness of using dual‐targeting in treating angiogenesis.^[^ ^86^ ^]^ A combination of an integrin α_v_β_3_ antagonist (cilengitide) and the TGFB‐R1 inhibitor (LY364947) was found to effectively suppress angiogenic activity of glioblastoma ToC.^[^ ^86^ ^]^ While cilengitide showed promising anticancer activity in preclinical and clinical trials^[^ ^318^ ^]^ the combination of cilengitide and LY364947 has yet to be tested in vivo. Thus, the CoC can potentially serve as a preclinical testing platform to predict clinical results and save time, costs, and resources.

##### CXCR2 Targeting

The efficacy of a CXCR2‐blocking antibody in preventing breast cancer metastasis to bone was studied in a bone OoC platform.^[^ ^87^ ^]^ Because CXCR2 is an important factor in promoting the colonization of breast cancer cells in bone cells, this targeting agent was highly effective in suppressing extravasation and metastasis.^[^ ^87^ ^]^ However, the CXCR2‐blocking antibody used is not a fully developed cancer therapeutic; thus, no animal studies or clinical trials can support these results. Future validation studies are needed to ensure the efficacy of the CoC for TCT development and testing.

##### MMP targeting

A Colorectal CoC proved effective in testing the efficacy of Marimastat, an MMP targeting agent, in treating colorectal cancer and preventing metastasis to liver cells.^[^ ^88^ ^]^ Indeed, Marimstat inhibited tumor migration by blocking MMP functions, which was validated in early in vivo animal studies.^[^ ^319^, ^320^ ^]^ However, Marimstat demonstrated adverse effects in clinical trials and has been discontinued.^[^ ^319^, ^320^, ^321^ ^]^ 5‐fluorouracil (5‐FU) was also found to be effective in suppressing tumor proliferation in this model; however, this was not a targeted therapy.^[^ ^88^ ^]^ While the colorectal CoC captured the anti‐metastatic effect of Marimstat, it failed to uncover its adverse, off‐target effects. Nevertheless, the colorectal CoC is a promising preclinical testing platform; future advancements and incorporating multi‐organ chambers in one chip can better predict clinical outcomes.

##### EGFR Targeting

Breast CoCs effectively tested the efficacy of Cetuximab, an EGFR‐targeting chemotherapeutic.^[^ ^89^ ^]^ Cetuximab was more effective in preventing migration of the more invasive cell line (MDA‐MB‐231) with higher EGFR expression, indicating efficient targeting. Furthermore, cetuximab also reduced the expression of other important EMT factors, including vimentin, enhancing its efficacy.^[^ ^89^ ^]^ Compared to animal studies on cetuximab's targeting efficacy,^[^ ^322^, ^323^, ^324^ ^]^ the breast CoC demonstrated similar results, indicating good accuracy of the platform in recapitulating in vivo outcomes. However, cetuximab has passed clinical trials and is FDA‐approved for colorectal cancer treatment only, not breast cancer.^[^ ^325^ ^]^ Future studies are needed to further validate the breast CoC model as a preclinical platform; however, compared to animal studies and clinical results, the CoC shows great promise.

#### 5.2.2 Testing Drugs Utilizing Triggered Targeting

##### Hypoxia‐Triggered Targeting

A liver‐lung MoC metastasis model proved effective in evaluating three hypoxia‐dependent chemotherapeutic agents targeting HIF‐1α: SYP‐5, IDF‐11774, and tirapazamine (TPZ).^[^ ^90^ ^]^ The unique two‐organ MoC design facilitated off‐target toxicity studies, where IDF‐11774, although effective on lung cancer cells, had harmful cytotoxic impacts on healthy liver cells in the MoC platform.^[^ ^90^ ^]^ IDF‐11774 is in an ongoing phase‐1 clinical study on colorectal cancer and has yet to report adverse toxic effects.^[^ ^326^, ^327^, ^328^ ^]^ Thus, further validation of results in vivo is needed to understand the CoCs potential as a preclinical platform to evaluate TCT safety and efficacy. On the other hand, a brain CoC model platform was used to develop and test a novel hypoxia‐targeting agent.^[^ ^91^ ^]^ The use of O_2_‐releasing microparticles to target hypoxia in tumor microenvironments was found to effectively suppress drug resistance induced by hypoxia. This targeted therapeutic agent was also effective in treating laryngeal cancer, as demonstrated in a CoC model.^[^ ^91^ ^]^ However, the hypoxia‐triggered chemotherapeutic is still recent, and further validation in vivo is needed to evaluate the CoCs efficacy as a preclinical testing platform.

##### Acidity‐Triggered Targeting

Using CoC, acidity‐triggered targeting was investigated, showing that calcium carbonate NPs (CaCO_3_ NPs) can raise the pH of tumor microenvironments to physiological pH, reducing cancer growth and inhibiting metastasis in a breast (MDA‐MB‐231).^[^ ^92^ ^]^ This ToC model was distinguished in its design, where both control and experimental setups were contained in one device, and precise control of pH parameters, among others, was easily permitted. These design parameters allowed for effective testing of nano‐CaCO_3_ and can be used to test other pH‐triggered chemotherapeutic agents.^[^ ^92^ ^]^ CaCO_3_ NPs are still early in development, with no extensive animal testing.^[^ ^329^ ^]^ Hence, CoCs may help in expatiating the clinical translation of the TCT.

#### 5.2.3 Testing Targeting Drugs with Non‐defined Mechanisms

A lung CoC demonstrated the targeting efficacy of a novel anticancer tryptophan‐rich peptide P1 (ACP) in inhibiting lung cancer tumor growth.^[^ ^93^ ^]^ Although the ACP was found to be highly selective to lung cancer cells with little impact on normal human cells, the exact mechanism behind the targeting efficacy of this peptide was not studied.^[^ ^93^ ^]^ Further studies using the CoC can provide a deeper insight into the targeting mechanism, explaining ACP's efficacy and predicting in vivo results. Moreover, a glioblastoma CoC proved effective in investigating the targeting efficacy of resveratrol (Res), an anti‐invasion agent, and combinations of Res and temozolomide (TMZ), where combinations of Res and TMZ were found to be more efficient in suppressing tumor invasion and proliferation by reducing MMP‐2 expression.^[^ ^94^ ^]^ Similar to the previous study, the exact targeting mechanism is unclear, and further research is needed to better understand them.^[^ ^94^ ^]^ Future animal studies can further validate these results.

Although the results of these studies are promising, more studies on the use of single‐ and multi‐organ CoCs for developing and testing targeted therapies are required to expedite the clinical translation of developed therapies following in vitro testing.

### 5.3 Testing Targeted, Encapsulated Chemotherapeutics

Designing chemotherapeutic agents that specifically target tumors is one effective method for developing targeted cancer therapy. Another common method is designing and synthesizing targeted drug delivery systems (DDSs) that encapsulate existing treatments and deliver them safely to the target site, reducing the side effects of chemotherapy. Similar to targeted chemotherapeutics, targeted drug delivery systems build on knowledge obtained after studying the tumor microenvironment and identifying potential targets, as discussed earlier. Cancer OoCs and MoCs are useful in studying and testing targeted drug delivery systems utilizing passive, active, and triggered targeting modalities. This section will herein explore these CoCs.

#### 5.3.1 Testing DDSs Utilizing Passive Targeting

An important factor to consider and understand in targeted drug delivery is the transport properties, accumulation, size parameters, and other factors related to passive targeting and the EPR effect. These factors can be studied using CoC platforms, as was done in^[^ ^69^, ^96^, ^251^ ^]^. Diffusivity, a key transport property impacting the delivery of MCF‐7 breast tumors, was successfully recapitulated in a breast ToC platform employed to study stromal‐ECM interactions.^[^ ^251^ ^]^ Dextran labeled with fluorescein isothiocyanate (FITC) was used to determine diffusivity in normal fibroblasts, cancer‐activated fibroblasts, and activated cell microtissues, where a significant difference in diffusivity was detected between normal and cancerous cells due to differing ECM composition and organization. This further impacts the interstitial resistance faced by drug carriers.^[^ ^251^ ^]^ Such detailed diffusivity studies are difficult to perform in vivo, requiring advanced imaging techniques.^[^ ^330^ ^]^ Thus, this breast cancer OoC platform is an effective model for testing drug delivery to the target site and transport properties.

Furthermore, a vasculature‐on‐a‐chip platform was used to decipher the EPR effect.^[^ ^96^ ^]^ Although widely accepted, the mechanism of EPR and its link to drug delivery is still not fully understood.^[^ ^96^, ^331^ ^]^ The leaky vasculature and ECM of tumor environments were recapitulated using SKOV3 ovarian cancer cells and HUVECs in a chip.^[^ ^96^ ^]^ The model was made to only allow 20‐kDa dextran to pass through while blocking 70‐kDa dextran to mimic in vivo conditions; then, the transportation of two types of NPs was investigated: soft pegylated liposomes (PEG‐Lip) and rigid poly(ethylene glycol)/poly(lactide‐*co*‐glycolide) NPs (PEG‐PLGA‐NPs). The transport of both carrier systems in the ToC model was relatively slow due to the rigid and dense ECM and endothelial barrier, while their extravasation and accumulation depended on different factors, including size and shape. Meanwhile, the rigidity of the NPs has little significance on accumulation in the ToC model. Subsequent animal studies further confirmed the insignificance of NP rigidity in transport and accumulation.^[^ ^96^ ^]^ Agreement in results obtained from the CoC platform and animal studies highlights the accuracy and efficacy of CoCs in testing targeted drug delivery systems.

A breast CoC further demonstrated the importance of NP size in passive targeting using gold nanoparticles (AuNPs).^[^ ^69^ ^]^ Importantly, the study highlights the significance of size, as NPs of 110 nm and above have poor retention and accumulation at the target site. Interestingly, these results were validated in vivo, where mice studies found enhanced accumulation of 50 nm NPs but poor retention for 160 nm NPs.^[^ ^69^ ^]^ These results stipulate that drug delivery systems should be designed to retain sizes below ∼100 nm for optimal transport and accumulation. More importantly, the close agreement between results obtained from CoCs and in vivo animal models proves the efficacy of CoCs as preclinical platforms for developing and testing passively targeted DDSs. Overall, the rich findings obtained from all these studies convey the benefits and efficacy of CoC platforms in demonstrating the importance of understanding passive‐targeting factors, including ECM composition, interstitial resistance, diffusivity, and NP properties, to ensure efficient transportation and drug delivery to the target site.

CoC platforms have also proven useful in investigating the efficacy of cancer treatment using encapsulated or free chemotherapeutics.^[^ ^68^, ^227^, ^332^ ^]^ For example, a co‐culture of kidney cancer and healthy liver cells in a single‐chamber CoC was used to test the efficacy of free 5‐FU compared to 5‐FU encapsulated in PLGA‐PEG‐NPs.^[^ ^332^ ^]^ The 5‐FU delivered by the NP was more effective and cytotoxic towards Caki‐1 kidney cancer cells in liver microenvironments than free 5‐FU treatment.^[^ ^332^ ^]^ PLGA‐PEG‐NPs loaded with 5‐FU have yet to be tested in animal models; however, other in vitro studies on cell cultures have shown similar results when loaded with different chemotherapeutics.^[^ ^333^ ^]^ Animal studies are needed to validate the results obtained from the CoC further. Similarly, an osteosarcoma CoC demonstrated lipid‐methotrexate NPs’ more rapid internalization at osteosarcoma tumor sites as compared to free methotrexate, and more significant cytotoxicity was observed when using lipid‐methotrexate NPs at the same concentration of free methotrexate.^[^ ^68^ ^]^ Conversely, colorectal CoC platforms were used to study the efficacy of CMCht/PAMAM dendrimer NP encapsulating gemcitabine (GEM), a cytotoxic chemotherapeutic agent.^[^ ^227^ ^]^ Dendrimer‐GEM NPs had greater cytotoxic efficacy and more rapid penetration into the tumor microenvironment, thus conveying the significance of drug delivery in targeted chemotherapy.^[^ ^227^ ^]^ All CoCs above proved convenient in testing encapsulated therapeutics and evaluating their efficacy in transporting drugs for effective tumor penetration.^[^ ^68^, ^227^, ^332^ ^]^ Further validation with animal models and subsequent clinical trials can help improve CoC designs for more accurate and clinically relevant results.

#### 5.3.2 Testing DDSs Utilizing Active Targeting

Active targeting is commonly used in the design of numerous drug delivery systems, and has been tested using different CoCs.^[^ ^69^, ^71^, ^80^, ^95^, ^96^, ^97^ ^]^

##### Transferrin

A breast CoC was effective in capturing the efficacy of transferrin targeted‐AuNPs (Tf‐AuNPs), where Tf improved accumulation at the tumor site in the CoC chip by 15 folds.^[^ ^69^ ^]^ In animal studies, the Tf‐targeted AuNPs exhibited a deeper penetration compared to the pegylated, non‐targeted AuNPs, which agrees with the CoC results and indicates the efficacy of Tf targeting and the accuracy of CoC platforms. However, although the targeting efficacy results from the CoC and in vivo experimentations agreed, targeting efficacy and penetration depth were more significant and effective in the CoC models, where the mice showed less significant findings.^[^ ^69^ ^]^ This discrepancy in the impact of targeting is crucial and could be due to variability in animal models.^[^ ^69^ ^]^ Further studies are needed to better understand this discrepancy in results to improve upon existing CoC accuracy.

##### VEGF

Liver (HepG2), lung (A549), and colorectal (SW620) vasculature‐on‐a‐chip cancer models were effective in testing VEGF‐ and VEGFR‐targeting siRNA‐mesoporous silica NPs (MSNs) as targeted anti‐angiogenesis therapeutics.^[^ ^80^ ^]^ siVEGFR/MSN was found to have a greater anti‐angiogenic influence on the ToCs and inhibited tumor growth, thus demonstrating high targeted delivery of the targeted therapeutic agent siVEGFR using MSN.^[^ ^80^ ^]^ These results were validated in animal models^[^ ^80^ ^]^ indicating CoC accuracy as a preclinical platform for TCT development and testing.

##### FA & Dual FA‐TAT

An ovarian CoC platform was useful for studying the targeting efficacy of FA‐ cell‐penetrating peptide (TAT) modified liposomes loaded with paclitaxel (PTX), compared to PEG‐, FA‐, and TAT‐liposomes.^[^ ^71^ ^]^ FA‐TAT‐liposomes exhibited the greatest accumulation and internalization at the tumor site, a higher targeting efficacy as compared to the other targeting moieties used, and the highest cytotoxicity when loaded with PTX. Efficacy decreased from TAT‐Lip to FA‐Lip, and PEG‐Lip, respectively.^[^ ^71^ ^]^ Compared to animal studies, similar results were obtained with FA‐TAT liposomes having a higher accumulation at prolonged treatment times. However, less accumulation was observed in vivo compared to the CoC for shorter treatment times. This discrepancy is attributed to the more complex in vivo microenvironment in animals^[^ ^71^ ^]^ compared to the simpler CoC platform, indicating the need to consider treatment times when using CoCs as preclinical testing models. Furthermore, increased flow rates correlated with higher resistance to treatment, an important factor that must be considered when designing such drug delivery systems for targeted therapy.^[^ ^71^ ^]^ Thus, the ovarian CoC proved effective in testing targeted drug delivery systems and mimicking in vivo conditions. Further validation can be achieved through future clinical studies.

Triple‐negative (FLORα overexpressed) and non‐triple negative breast CoCs were also effective in demonstrating the high targeting efficiency of doxorubicin (DOX)‐loaded FA‐modified carbon dot (FA‐PEG‐CD/DOX) NPs.^[^ ^95^ ^]^ Validation using animal studies is needed to understand the breast CoCs accuracy in recapitulating in vivo microenvironments. A vasculature‐on‐a‐chip platform was also used to test FA‐targeted liposomes and FA‐targeted PEG‐PLGA‐NPs, and the model was validated using in vitro 2D cultures and 3D spheroids, as well as in vivo in mice models.^[^ ^96^ ^]^ Although FA‐targeting caused significant increases in accumulation in 2D cultures and 3D spheroids, the same FA‐targeted DDSs showed no statistically significant difference in accumulation compared to non‐targeted carriers when tested using the CoC model and animal studies.^[^ ^96^ ^]^ Interestingly, in vivo findings matched the CoC models, proving they better recapitulate in vivo physiological responses. Future advancements in CoC models can better enhance their efficacy in predicting in vivo and clinical outcomes.

##### HA

Another breast CoC was advantageous in evaluating the targeting efficacy of HA‐NPs loaded with DOX.^[^ ^97^ ^]^ Again, encapsulated DOX delivery was found to be more effective than free DOX delivery due to the enhanced penetration caused by the targeted HA NPs.^[^ ^97^ ^]^ No animal studies were conducted to further validate results; thus, future validation studies are needed to comprehensively understand the CoC's accuracy. Nonetheless, CoC systems have been proven effective in studying actively targeted DDSs.

#### 5.3.3 Testing DDDs Utilizing Triggered Targeting

##### Internal Triggering

CoC platforms have been effective in recapitulating the internal properties of tumor microenvironments, thus having high potential as a preclinical platform for testing targeted drug delivery through internal triggering mechanisms.^[^ ^98^, ^99^, ^100^ ^]^

For instance, an ovarian CoC platform recapitulated the hypoxic tumor microenvironment effectively and was useful in studying the efficacy of hypoxia‐sensitive micellular NPs encapsulating siRNA and DOX.^[^ ^98^ ^]^ The hypoxia‐sensitive micelles were composed of PEG‐azobenzene‐ polyethylenimine‐dioleoylphosphatidylethanolamine (PAPD); when treating human ovarian CoCs with PAPD micelles, the hypoxic environment caused the PEG layer to shed, which subsequently enhanced drug delivery and internalization into the tumor.^[^ ^98^ ^]^ Studies in mice further supplemented these results and conveyed the efficacy of hypoxia‐targeted drug delivery.^[^ ^98^ ^]^

Another internal property utilized for the design of targeted drug delivery systems is pH.^[^ ^99^ ^]^ A dual heart‐breast MoC system was developed to study cardiotoxicity from DOX during breast cancer treatment, as well as investigate the efficacy of pH‐triggered drug delivery in reducing cardiotoxic impacts. Graphene‐based yolk‐shell magnetic nanoparticles (GYSM‐NPs) were loaded with DOX and used for pH‐targeted drug delivery in the MoC chip, where DOX release increased with decreasing pH of the tumor microenvironment.^[^ ^99^ ^]^ This indicates the high targeting efficacy of the drug carrier. Furthermore, lower cardiotoxic effects and lower proliferation were observed in cardiac tissue when treated with GYSM‐NP/DOX system, as compared to free DOX, indicating more selective delivery to the tumor environment.^[^ ^99^ ^]^ Further validation of these results using animal models is needed to better understand CoC accuracy as preclinical testing platforms.

Similarly, an alveolus‐epithelium CoC accurately recapitulated the acidic pH of the tumor microenvironment and demonstrated the efficacy of pH‐sensitive ZnO‐quantum dot (ZnO‐QD) loaded human serum albumin (HSA) NPs.^[^ ^100^ ^]^ Non‐invasive optical pH sensors added further value to the CoC, as continuous pH monitoring and control were achieved. Furthermore, a built‐in indium titanium oxide‐based TEER sensor was incorporated for cytotoxicity studies.^[^ ^100^ ^]^

Thus, single‐ and multi‐organ CoCs are valuable platforms for evaluating both hypoxia and pH‐triggered drug delivery systems.^[^ ^98^, ^99^, ^100^ ^]^

##### External Triggering

CoC models have also been useful in studying externally triggered targeted drug delivery systems. For example, a vascularized breast CoC demonstrated the efficacy of near‐infrared (NIR) radiation‐activated NPs.^[^ ^101^ ^]^ These NPs consist of an inner fullerene core within a mesoporous silica matrix and are surrounded by an outer lipid layer encapsulating DOX and green indocyanine.^[^ ^101^ ^]^ The transparent CoC design allowed for NIR irradiation and triggered drug release, subsequently reducing cell viability and inhibiting tumor growth.^[^ ^101^ ^]^ These results were consistent with in vivo animal studies,^[^ ^101^ ^]^ indicative of the CoC accuracy in reproducing in vivo microenvironments. Moreover, another breast CoC model was used to evaluate the targeted delivery of a photosensitizer agent to the tumor site for subsequent photodynamic therapy (PDT) and tumor eradication.^[^ ^102^ ^]^ The photosensitizer precursor 5‐aminolevulinic acid (5‐ALA), encapsulated in Au‐NPs, demonstrated highly improved PDT treatment efficacy and uniformity.^[^ ^102^ ^]^ Similar to the previous CoC, the transparent, PDMS‐based platform allowed for effective photodynamic therapy and testing of externally triggered chemotherapeutics.^[^ ^102^ ^]^ However, further testing using animal models is needed to validate results and highlight the accuracy of CoCs as preclinical platforms for TCT development and testing.

**Figure 6:**
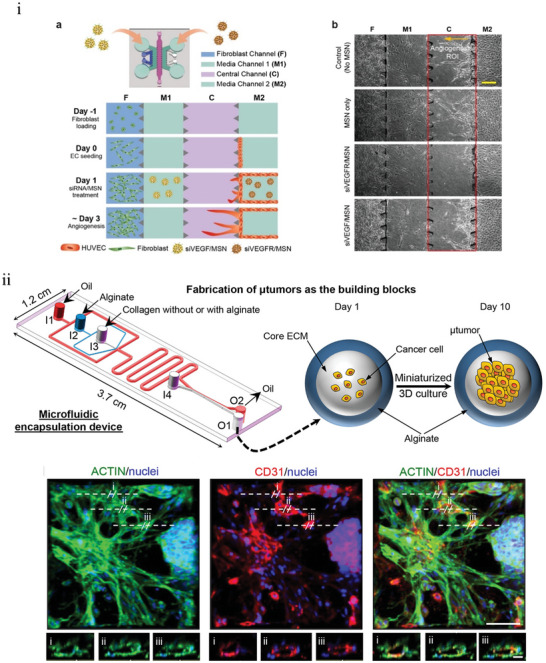
The use of OoCs and MoCs in the investigation and evaluation of targeted drug delivery systems through passive, active, and triggered targeting strategies. i) In vitro regulation of cancer angiogenesis using a 3D microfluidic platform using MSNs loaded with or without siVEGF or siVEGFR. Adapted from^[^ ^80^ ^]^ with permission from ACS Publications. ii) A microfluidic device for encapsulating cancer cells in core–shell microcapsules, which form microtumors after incubation for 10 days, and testing NIR activated NPs. Staining for ACTIN filament, CD31, and cell nuclei showed extensive vascularization of the 3D tumors. Reproduced with permission.^[^ ^101^ ^]^ Copyright 2017, ACS Publications.

Overall, various CoC platforms demonstrated high accuracy and efficacy in testing targeted drug delivery systems via different modalities (passive, active, triggered) and understanding their mechanisms (**Figure** **6**). This highlights the great potential of CoCs as novel, accurate preclinical platforms for TCT development and testing (see **Table** **3**).

**Table 3:** Summary of targeted drug delivery studies using organ‐on‐chips and multi‐organ‐on‐chips.

Drug Delivery System	Agent encapsulated	OoC (target site)	Targeting mechanism	Main results	Reference
Fluorescein Isothiocyanate (FITC)‐labeled Dextran	None	Breast CoC (MCF7 cells)	Passive targeting	– Studied differences in transport properties, including diffusivity and interstitial resistance, between cancerous and normal microtissues. – Transport properties are important factors to consider when designing targeted drug delivery systems.	[251]
Dextran (20‐kDa and 70‐kDA)	None	Ovarian cancer vasculature‐on‐a‐chip (SKOV3 cells)	Passive targeting (enhanced permeability and retention (EPR))	– 70‐kDA dextran carriers could not pass through the leaky vasculature of ovarian cancer, while 20‐kDA dextran managed to pass through. – The size of the drug carrier is important for targeted drug delivery.	[96]
Soft pegylated liposomes and rigid poly(ethylene glycol)/poly(lactide‐*co*‐glycolide) (PLGA‐PEG)‐ and Folic Acid (FA) FA‐liposomes	None	Ovarian cancer vasculature‐on‐a‐chip (SKOV3 cells)	‐ Passive targeting ‐ Active targeting (biological)	– PLGA‐PEG‐liposome accumulation depended on size and shape, while rigidity was not as significant in the CoC model. – Extracellular matrix composition impacts drug carrier transport – There was no significant difference in the accumulation of passive (PLGA‐PEG‐) and active (FA‐) liposomes in the CoC model and in in vivo studies.	[96]
PEG‐gold nanoparticles (PEG‐AuNPs)and transferrin (Tf) ‐AuNPs	None	Breast cancer CoC (MDA‐MB435)	‐ Passive targeting ‐ Active targeting (biological)	– NP size is especially important where NPs above 110 nm have a low accumulation at the target site. – Active targeting using Tf improved accumulation and retention at the tumor site. – Flow dynamics in vasculature impacted both passive and active targeting and accumulation, but penetration was unaffected.	[69]
siRNA mesoporous silica NPs (MSN)	‐ Small interfering vascular endothelial growth factor (siVEGF) ‐ siVEGF receptor (siVEGFR)	Vasculature‐on‐a‐chip of lung (A549), liver (HepG2), and colorectal (SW620) cancer.	Active targeting (biological)	– Both si‐VEGF/mesoporous silica NPs (MSNs) and si‐VEGFR/MSN targeted angiogenesis, while siVEGFR/MSN showed a higher targeting efficacy and anti‐angiogenic effect. – HepG2 CoC was the most impacted by siVEGFR therapy, unlike A549 and SW620 CoCs had relatively lower or insignificant changes.	[80]
PEG‐, folic acid (FA)‐, cell penetrating peptide (TAT)‐, and FA‐TAT‐Liposome (Lip)	Paclitaxel (PTX)	Ovarian cancer CoC (SKOV3)	Active targeting (biological)	– Most effective with highest accumulation was FA‐TAT‐Lip, followed by TAT‐, FA‐, and PEG‐Lip. – Multi‐target drug carriers have greater success. – High flow rates lead to high resistance to therapy.	[71]
FA‐PEG‐Carbon dot (CD)	Doxorubicin (DOX)	Breast cancer CoC (BT549 and T47D)	Active targeting (biological)	– FA‐PEG‐CD/DOX had a greater impact on BT549 cells compared to T47D cells, meaning it targets triple‐negative breast cancer.	[95]
Hyaluronic acid (HA)‐NPs	DOX	Breast cancer CoC (MCF‐7, MDA‐MB‐231, SUM‐159PT)	Active targeting (biological)	– Targeted drug delivery was achieved and cell proliferation was impeded. – HA‐NP/DOX had the most significant impact on MCF‐7 cells, while some cell survival was observed in MDA‐MB‐231 and SUM‐159PT cells – showing varying targeting efficacy in different cancer cell lines.	[97]
poly(lactide‐*co*‐glycolide)‐ poly(ethylene glycol) nanoparticles (PLGA‐PEG‐NP)	‐ 5‐fluorouracil (5‐FU) ‐ Coumarin	Metastasis‐on‐a‐chip (kidney cancer and liver cell co‐culture, Caki‐1 and HepLL)	Passive targeting	– Although using passive targeting, 5‐FU loaded PLGA‐PEG‐NP was more effective in treating metastasis than free 5‐FU. – Delivery of 5‐FU and Coumarin‐6 is better when encapsulated in liposomes.	[332]
Lipid‐NPs	Methotrexate	Osteosarcoma CoC (U‐2 OS cells)	Passive targeting	– Greater and faster internalization was achieved with methotrexate‐lipid‐NPs compared to free methotrexate and higher cytotoxic effect.	[68]
CMCht/ PAMAM dendrimer	GEM	Colorectal cancer CoCs (HCT‐116)	Passive targeting	– Dendrimer‐GEM NPs are more cytotoxic with greater penetration compared to free GEM.	[227]
PEG‐azobenzene‐ polyethylenimine‐dioleoylphosphatidylethanolamine (PAPD) micelles	siRNA and DOX	Ovarian cancer CoC (2780 ADR cells)	Triggered targeting (internal – hypoxia)	– The hypoxia‐sensitive micelles effectively released DOX and siRNA at the tumor site, causing high cytotoxicity.	[98]
Graphene‐based yolk‐shell magnetic (GYSM) NP	DOX	Heart‐breast cancer MoC (SK‐BR‐3 breast cancer cells)	Triggered targeting (internal – pH)	– GYSM‐NP/DOX exhibited greater cytotoxicity and effective targeting due to lower cardiotoxicity compared to free DOX.	[99]
Human serum albumin (HSA) NPs	ZnO‐quantum dots (QD)	Alveolus‐epithelium lung cancer CoC (A549)	Triggered targeting (internal – pH)	– ∼90% ZnO‐QD release achieved at pH = 5.0, while minimal release observed at pH = 7.4. – ∼80% cytotoxicity achieved at a 50 µg/mL dose indicates effective targeting and antitumor properties.
Lipid (L), fullerence (C60), silica (S), DOX (D), and indocyanine green (ICG or I), (LC60S‐DI) NPs.	DOX and indocyanine green	Breast cancer vascular CoC (MCF‐7)	Triggered targeting (external – near‐infrared radiation (NIR))	– High targeting efficacy was observed with high cytotoxicity when triggered with NIR.	[101]
Au‐NPs	5‐aminolevulinic acid (5‐ALA)	Breast cancer CoC (MCF‐7)	Triggered targeting (external – light)	– Au‐NP assisted delivery of 5‐ALA with external light stimulation had a greater photodynamic therapy efficacy and cytotoxic impact, while 5‐ALA alone exhibited lower anti‐tumor efficacy.	[102]

## 6 Current Challenges and Future Directions

The transition from 2D to 3D cell cultures has significantly improved drug delivery and targeting experiments. At the same time, it potentially lowered the failure rate of preclinical and clinical studies.^[^ ^77^ ^]^ CoCs have substantially evolved as an alternative culture model, which proved to be promising in developing and testing targeted chemotherapeutics.

### 6.1 Current Challenges

Although CoC systems have improved experimental prediction and advanced targeted chemotherapeutic research by better mimicking physiological conditions compared to 2D cell cultures,^[^ ^75^, ^236^, ^334^ ^]^ in vivo tumor microenvironments are more complex. Hence, CoCs are not fully capable of replicating biological conditions and structures in vivo, and many challenges remain to be resolved. Achieving a perfect biomimicking system involves a high degree of complexity and implementing such a high level of complexity is nearly impossible with CoCs. Inclusion of vasculature, proper stroma composition (e.g., fibroblasts), immune cells, and other microenvironment components, their relative locations, activity, gradients, etc. impose limits on CoC capability to completely reproduce the microenvironment seen in native tumors.^[^ ^207^ ^]^ However, it offers the possibility to dissect these events and structures to analyze effects on a single or few types of cells in a very focused manner that can help to elucidate a better understanding of targeting process, mechanism, safety, and efficacy.^[^ ^65^, ^69^, ^71^ ^]^ This understanding is achieved in a way better than that which can be achieved using 2D cultures and in a way more representative of what can occur in the human body than what can be observed using different species (animal studies).^[^ ^65^, ^75^ ^]^ Although current systems proved a certain degree of reliability^[^ ^75^ ^]^ and can be used as such for drug testing and development, there is a space and need for improvement and development of complex multi‐cell type systems to enable the development of systems closer to the human body. The governing outcome judgment will be based on how target drugs mimic observations seen in humans and in the clinic.

Among the challenges related to the use of CoC systems in targeting studies are the type of materials used in their development, scaling issues, difficulty in testing external triggering modalities, reproducibility and standardization, and psychological resistance.

Recent studies have reported the influence of some materials used on the adsorption of the drugs' polymer‐based microchips.^[^ ^212^, ^214^, ^215^, ^335^ ^]^ For example, PDMS can absorb hydrophobic small‐molecule medicines, reducing their bioavailability and resulting in inaccurate drug‐response predictions.^[^ ^212^, ^214^, ^215^ ^]^ Further studies revealed other small molecule drugs with an affinity to non‐specific binding have also been absorbed by PDMS, regardless of hydrophobicity.^[^ ^215^, ^336^ ^]^ This poses a significant challenge in measuring targeting efficacy, as many targeted chemotherapeutics are small‐molecule drugs.^[^ ^18^ ^]^ Since drug absorption into PDMS is an equilibrium process, portioning coefficients can be computed to account for reductions in drug concentrations.^[^ ^212^ ^]^ Optimizing surface area‐to‐volume ratios of the CoC channels and chambers can also minimize drug absorption.^[^ ^212^ ^]^ In addition, surface coatings, such as sol‐gel treatments^[^ ^220^ ^]^ and lipophilic coatings,^[^ ^215^ ^]^ and alternative materials, including polyurethane elastomers,^[^ ^217^ ^]^ thermoplastic elastomers, polystyrene,^[^ ^218^ ^]^ PMMA, and polycarbonates^[^ ^220^ ^]^ have been introduced as potential solutions. Hydrogels and paper have also been introduced as alternatives but are limited in their optical transparency, sterilization process, and leaching risk.^[^ ^216^ ^]^ Glass is another alternative that has shown great promise in the development of CoCs, using lasers for welding and channel formation.^[^ ^337^, ^338^ ^]^ However, further research is needed to ensure the suggested materials are suitable for cell cultures while being flexible, optically transparent, and permeable to gasses, among other properties.^[^ ^216^, ^217^, ^218^, ^220^ ^]^

While the miniaturized size of CoC systems is beneficial in reducing material consumption costs, it introduces a new challenge: scaling.^[^ ^335^ ^]^ This is because the number of cells cultured, flow rates, medium concentrations, and drug dosages used are far away from those seen in real tissues.^[^ ^339^ ^]^ With MoC platforms, scaling limitations become more prominent as there is still no clear understanding of the effect of scaling on interacting organs.^[^ ^340^ ^]^ Disproportionate size ratios between different organs in an MoC cancer system can affect physiological processes and interorgan interactions, reducing the model's authenticity in mimicking in vivo conditions.^[^ ^339^, ^340^ ^]^ Different scaling approaches have been introduced to guide CoC and MoC design and construction, including allometric scaling, functional scaling, and scaling by residence time and organ mass; these have been reviewed and critiqued.^[^ ^339^, ^340^ ^]^ In addition, when testing targeted therapeutics using CoCs and MoCs, the translation of results must be recalculated and adjusted. With current, rapid advances in machine learning and artificial intelligence, scaling calculations and challenges could be tackled using artificial intelligence.^[^ ^341^ ^]^

CoCs often consist of multiple layers to better mimic in vivo conditions by incorporating channels for media flow, cell cultures, gas gradients, and other features.^[^ ^75^ ^]^ However, such multilayer designs can make it difficult to study the efficacy of targeting agents utilizing external triggering modalities, such as ultrasound, magnetic waves, light, and heat. Because CoCs are often constructed using PDMS, ultrasound application should not pose a significant challenge as PDMS is an acoustically transparent medium.^[^ ^342^ ^]^ Nonetheless, only a few studies have investigated ultrasound‐triggered therapies using OoCs.^[^ ^343^, ^344^, ^345^ ^]^ For example, focused ultrasound‐triggered microbubble treatment's therapeutic impact and underlying mechanism were studied successfully using an optically transparent blood–brain barrier OoC.^[^ ^345^ ^]^ Another microfluidic platform adopted a vertical design with four layers to allow for enhanced visualization, acoustic transparency, and localization of applied ultrasound thermal and mechanical impacts.^[^ ^344^ ^]^ In this platform, drug release from thermosensitive liposomes was achieved by the thermal effects of focused ultrasound.^[^ ^344^ ^]^ However, a significant challenge that often arises in ultrasound studies is incident wave reflection, thus necessitating submersion in water to minimize reflection.^[^ ^343^ ^]^ Submersion in water can be challenging when using CoCs, but it is possible – as demonstrated by the opti‐cells ^[^ ^343^ ^]^ for ultrasound‐facilitated drug delivery studies. Further optimization is needed for more accurate and relevant studies on ultrasound‐triggered chemotherapeutic systems (see Figure 6).

Similarly, studying magnetic triggering using CoCs requires using materials transparent to magnetic waves to avoid interferences. Magnetic resonance imaging is one method of magnetic stimulation CoCs, as demonstrated in a breast ToC model developed to study superparamagnetic iron oxide NPs as theranostic agents.^[^ ^346^ ^]^ Meanwhile, using special magnets on the chip, a more straightforward microfluidic system lacking biomimetic properties proved valuable in studying NP's responses to magnetic field stimulation.^[^ ^347^ ^]^ Although successful in understanding the impact of magnetic triggering, the platform was simple with no biological matter; thus, in vivo translation of such results requires the use of more relevant biomimetic CoCs.^[^ ^347^ ^]^ Nonetheless, these models can be used as preliminary data to build off from and design more effective platforms to study externally triggered targeted chemotherapies.

Reproducibility is another significant challenge faced when testing targeting using OoCs. Although reproducible results have been achieved from OoC platforms in different labs,^[^ ^84^ ^]^ reproducibility is still a challenge that is faced, which also affects standardization. Building CoCs is a multi‐step process with various elements and variables that can make achieving reproducibility difficult.^[^ ^207^ ^]^ The greater the variability of each component, the greater the overall variability of the platform and the more difficult it is to obtain reproducible results.^[^ ^207^ ^]^ For example, modification of the surface of CoC microchannels is often irregular and time‐consuming, which can introduce some inconsistencies in the chip, thus affecting reproducibility and impacting the CoC lifetime.^[^ ^348^ ^]^ Introducing special coatings for microchannels can improve this by reducing irregularities.^[^ ^348^ ^]^ Furthermore, manual assembly of CoC platforms is associated with high‐user dependency, which introduces high variability. Similarly, technical difficulties, including bubble formation and contaminations, and intercell variability, especially when using primary cells, also heavily impact the accuracy and reproducibility of results obtained.^[^ ^207^ ^]^ The introduction of standardized protocols and the integration of automated processes, especially on the industrial scale, can significantly reduce variability between CoC chips, which in turn allows for more efficient and statistically verifiable targeting studies. Subsequently, this can also speed up the translation of targeted therapies into clinical studies because the testing platform (CoCs) would produce results of greater reliability and reproducibility. However, standardization of CoC and MoC platforms is a major challenge as it requires the standardization of all the various elements in these devices.^[^ ^207^ ^]^ Numerous organizations are currently studying the introduction of standardized protocols at different levels in OoC designs, including the European Commission Joint Research Centre, CEN, CENELEC,^[^ ^349^ ^]^ US working groups such as the National Institute for Standards,^[^ ^350^ ^]^ Innovative and Quality Microphysiological (IQ MPS) Affiliate,^[^ ^351^, ^352^ ^]^ the FDA^,[^ ^352^ ^]^ the 3Rs Collaborative,^[^ ^353^ ^]^ and Japanese Agency for Medical Research and Development (AMED) in Japan.^[^ ^354^ ^]^ Setting standards will majorly facilitate communication, characterization, and comparison of TCToC products, which will help accelerate their industrial production and clinical translation.

Ethical concerns regarding issues such as the use of human brain CoCs have risen as obstacles.^[^ ^355^, ^356^ ^]^ Researchers in Japan have delved into the possible legal status of brain organoids and discussed the possibility of considering brain organoids as “legal” humans with rights.^[^ ^357^ ^]^ At their current, premature state, legal and ethical concerns are minimal, and brain organoids are not considered legal or moral people.^[^ ^357^ ^]^ Since brain CoCs and MoC cancer models are similar to brain organoids in nature, similar ethical and legal concerns are likely to arise with future advancements in brain CoCs, posing a serious challenge. However, in light of current OoC advancements in the market^[^ ^358^ ^]^ and with the establishment of strict regulatory guidelines and standardized procedures by trustworthy organizations and by presenting the promising advantages of OoCs, it is envisioned that OoCs will face acceptance and will revolutionize and accelerate the drug development process.

### 6.2 Future Directions

High‐throughput screening is highly significant in expediting the drug development process by screening and testing multiple therapeutic agents in parallel under similar conditions.^[^ ^226^, ^359^ ^]^ A significant challenge is adapting high‐throughput automated screening in complex CoC platforms incorporating multiple cell types.^[^ ^359^ ^]^ For high‐throughput screening to be possible and accurate using CoCs, defined cultures need to be used with low variability to ensure reproducible results.^[^ ^226^ ^]^ While some developed CoC systems have successfully integrated high‐throughput screening of chemotherapies, these platforms often consist of monocultures or simple co‐cultures of the tumor microenvironment, which are less biologically relevant and representative of in vivo conditions.^[^ ^226^ ^]^ For instance, monoculture U87 spheroids were used to develop brain CoCs for high‐throughput screening of pitavastatin and irinotecan drug combinatorial with different concentrations to determine drug efficacy, define the optimal dosage, and study synergistic effects of the chemotherapeutic combination.^[^ ^270^ ^]^ Despite efficient and rapid high‐throughput screening, the brain CoC did not fully recapitulate the tumor microenvironment complexity observed in brain tumors, thus resulting in data with lower clinical relevance. CoCs with greater biomimicry are currently limited to low‐throughput due to difficulties adapting automation and high‐throughput screening tools for reliable, reproducible target identification and targeted therapy testing between laboratories.^[^ ^359^ ^]^

Future research is needed to balance and optimize CoC designs to be easily automated for high‐throughput screening of targeted therapeutics while also maintaining accurate, biological relevance by using complex but defined cell cultures. Recent research by Azizgolshani et al. ^[^ ^360^ ^]^ developed a novel OoC platform combining 96 miniature, standard plate size OoC devices of different organ cultures in one chip and integrating 384 electrical sensors and 192 controllable micropumps, all of which are programmable and easily automated; mono‐ and co‐cultures of different organs were used in individual devices, and real‐time optical imaging was adopted. The chip successfully performed multiple parallel experiments via high‐throughput screening tools.^[^ ^360^ ^]^ Thus, with further research, developing CoCs for high‐throughput screening of targeted cancer therapies is possible, and the establishment of such platforms will expedite the bench‐to‐bedside translation of targeted cancer therapies.

Another significant advancement in cancer research involves integrating organoids with OoCs, merging the benefits of these two bioengineering technologies. This integration enables researchers to study cancerous organoids in a controlled environment that mimics the blood flow and drug delivery dynamics in the tumor microenvironment. These hybrid systems mimic in vivo conditions, enabling detailed tumor studies and therapy responses without animal models, while precisely controlling the biochemical environment for improved disease modeling and drug testing accuracy.^[^ ^361^, ^362^ ^]^ The integration also facilitates studying systemic interactions between different organ chips, simulating tumor metastasis to distant organs.^[^ ^363^ ^]^ Personalized medicine and targeted therapeutics can also benefit from these hybrid systems because cells derived from individual patients can be used to develop personalized organoids, which can then be tested on chips to predict the most effective treatments for that specific patient, potentially revolutionizing personalized medical treatment. For example, Strelez et al. ^[^ ^364^ ^]^ developed a hybrid microfluidic OoC system incorporating patient‐derived colorectal cancer organoids, to study the effect of mechanical forces and neurotransmitter signaling on colorectal cancer. Using this system, they were able to identify GABAergic properties and the role of the GABA catabolism enzyme ABAT in colorectal cancer cell invasion, suggesting potential targets for drug development.

Despite its advantages, the integration of organoids with OoCs for cancer research is still premature and faces several challenges, such as the need to ensure that the organoids are compatible with the microfluidic systems used. This often requires precise control over factors like organoid size, placement, and the medium composition. The development of truly biomimetic biomaterials and the application of innovative biofabrication techniques are essential aspects to enhance the integration of organoids and OoCs, overcoming limitations related to the integration of 3D constructs into microfluidic devices.^[^ ^365^ ^]^ Scalability is another significant challenge, as maintaining the functionality and viability of integrated systems over extended periods is difficult, which is crucial for long‐term studies, e.g., assessing the cytotoxicity of cancer treatments. Recent advancements in this field aimed at addressing these challenges include microfluidic systems that can dynamically adjust to support the growth of organoids while maintaining precise control over the biochemical gradients necessary to simulate the tumor microenvironment accurately.^[^ ^366^ ^]^ Studies have also explored the use of bioprinting technologies to create more standardized organoids that can be easily integrated into OoC systems.^[^ ^367^ ^]^

There is also extensive research looking into creating more complex multi‐organ models that incorporate multiple types of organoids into a single chip: an approach that could revolutionize research on cancer metastasis and multi‐organ interactions during cancer progression and treatment. For example, Rajan et al. ^[^ ^368^ ^]^ developed a multi‐organoid system using a microfluidic device composed of interconnected chambers embedded with a hyaluronic acid hydrogel for 3D tissue organoid culture, enabling simultaneous drug efficacy and toxicity assessment. The preliminary setup consisted of three chambers designated for liver, cardiac, and lung organoids, where capecitabine (CAP) was introduced into the liver chamber, resulting in observed cytotoxic effects in the cardiac organoid due to the metabolite 5‐FU. In a subsequent model, additional chambers for endothelium, brain, and testes were incorporated, and the administration of the anticancer drug ifosfamide (IFO) via the liver organoid led to neurotoxic effects caused by its metabolites. Such multi‐organoid‐OoC systems would enhance our understanding of the disease and significantly improve the screening and evaluation of anti‐cancer drugs.^[^ ^369^ ^]^ Furthermore, combining these hybrid systems with artificial intelligence (AI) and high‐throughput screening expands its applications in cancer research and drug development. AI algorithms help analyze complex data from these models, offering insights into tumor behaviors and treatment responses, while high‐throughput platforms facilitate the rapid testing of potential treatments.^[^ ^370^, ^371^, ^372^ ^]^ Hence, the integration of organoids in cancer OoC platforms shows great promise for future TCT development and testing.

Although there have been improvements in the area of developing targeted chemotherapeutics using CoCs, to this day, preclinical trials using animal models are regarded as the gold standard, and translating the concept of OoCs into the clinical is still in the early stages. Testing targeted chemotherapeutics using CoCs generally yielded results that overall correlated with those obtained from animal studies.^[^ ^71^, ^80^, ^91^, ^96^, ^101^ ^]^ However, some discrepancies are noted between animal studies and CoCs, inhibiting the complete replacement of animal studies. For instance, significant tumor growth inhibition was observed within 24 hours in a CoC platform, while efficacy of targeted therapeutics was only observed after 48 hours in animal studies, showing the delayed significance of results.^[^ ^71^ ^]^ Delayed drug action can be attributed to the greater complexity of animals, as they represent whole organisms, unlike CoCs that only model the tumor site or a part of it.^[^ ^71^ ^]^ However, the relevance of these discrepancies remains questionable due to the inherent difference between animal species and humans.^[^ ^58^ ^]^ Although CoCs present a promising alternative, further developments in CoC and MoC design are needed to better represent clinical results. Even with future improvements, including high‐throughput screening, which is almost impossible to conduct in animal studies,^[^ ^373^ ^]^ ethical concerns and social resistance are predicted to challenge the use of CoCs as complete alternatives to animal studies. CoCs are more likely to serve as supplementary testing platforms alongside animal studies to obtain a comprehensive understanding of the efficacy of targeted chemotherapeutics, thus decreasing rates of animal testing, reducing costs, and more accurately predicting clinical outcomes.^[^ ^374^ ^]^

CoC platforms utilizing patient‐derived cells essentially represent individual patients, recapitulating their complex physiology and genetic makeup. This opens the possibility of conducting “clinical‐trials‐on‐a‐chip,” where individual CoCs represent individual patients in a clinical trial.^[^ ^194^ ^]^ However, fixed protocols are needed to produce reproducible CoCs that recapitulate complex patient genetic heterogeneity while also being easy to operate, that accommodate multiple organs to represent inter‐organ interactions and metastasis, and that include parallelization for rapid and accurate high‐throughput screening.^[^ ^76^ ^]^ Although challenging, clinical‐trials‐on‐a‐chip using MoC cancer models is highly possible. What further supports this is a recent study that developed the Ewing Sarcoma bone cancer – heart multi‐organ CoC platform to evaluate the efficacy of Linsitinib, a chemotherapeutic agent.^[^ ^77^ ^]^ Results from drug efficacy and toxicity studies on the multi‐organ CoC agreed with clinical trial results, while traditional preclinical testing methods (2D and animal studies) failed to reveal the cardiotoxicity of Linsitinib.^[^ ^77^ ^]^ Hence, if successfully developed, CoC devices could serve as a promising pre‐clinical model for evaluating developed targeted chemotherapeutics, better predicting clinical trial outcomes and decreasing the risk of harming patients and clinical trial failure. Furthermore, clinical‐trials‐on‐a‐chip using CoCs could be useful in the design of “targeted” clinical trials tailored to certain patient groups (based on ethnicity, genetic makeup, etc.).^[^ ^375^ ^]^ Patient cohort selection can be optimized so that only those who will most likely benefit from the chemotherapeutic proceed to the clinical trial; this can be determined via pre‐clinical, high‐throughput screening of targeted cancer therapies.^[^ ^76^, ^78^ ^]^ Nonetheless, only clinical studies can reveal comprehensive results regarding the true efficacy of developed targeted chemotherapeutics, and CoCs will most likely serve as a supplementary preclinical testing platform.

Developing personalized, targeted cancer therapies is of great importance in cancer therapy. Although useful in preliminary studies, immortalized cancer cell lines do not truly recapitulate in vivo tumor microenvironments in cancer patients.^[^ ^194^, ^226^ ^]^ Genetic profile variabilities are unaccounted for in immortalized cell lines, thus providing an incomplete picture of drug activity, efficacy, and toxicity.^[^ ^194^ ^]^ Thus, current research is shifting towards precision medicine, and CoCs have already proven to be unique platforms for modeling tumors of individual patients to study and test targeted cancer therapies.^[^ ^76^ ^]^ With the increased impacts of genetic and ethnic backgrounds on patients’ responses to cancer therapies, the need for personalized treatments will be critical soon.^[^ ^75^ ^]^ CoCs can be developed using cells from surgical biopsies, discarded tissues, adult stem cells, and induced pluripotent stem cells to establish effective, high‐throughput platforms for targeted, personalized cancer therapies.^[^ ^75^ ^]^ Current personalized CoCs are still preliminary models and face significant challenges, including short cell viability and reducing the lifetime of CoCs. Future research optimizing personalized CoC design and development is needed.

In addition to clinical‐trials‐on‐a‐chip, emerging OoC research has been looking into developing “human‐body‐on‐a‐chip” systems, which aim to comprehensively represent the majority of human function in a multi‐organ OoC platform.^[^ ^376^, ^377^, ^378^, ^379^, ^380^ ^]^ Although currently premature in nature, with future advances, integration of AI and automation, establishment of defined cultures, etc., these human‐on‐a‐chip platforms can essentially model a patient as a whole, allowing for deeper understanding and study of cancer mechanisms at the tumor site and its interaction with other organs.^[^ ^79^, ^375^ ^]^ These human‐body‐on‐a‐chip platforms incorporating cancerous organs will facilitate target identification and the development and testing of targeted cancer therapies, including toxicity studies, to reveal adverse side effects that could be absent in animal studies.^[^ ^76^, ^226^, ^375^, ^379^ ^]^ Up to 10 organs have been modelled in MoC cancer models.^[^ ^80^, ^88^, ^90^, ^99^, ^211^, ^237^, ^238^, ^239^, ^240^, ^241^, ^242^, ^243^, ^244^, ^245^, ^246^, ^247^ ^]^ Although premature, future research and advances are predicted to generate more accurate and representative human‐body‐on‐a‐chip cancer models for targeted cancer therapy development and testing.

Targeted cancer therapy development using CoCs can potentially be elevated by incorporating aspects of space medicine and gravitational biology. Microgravity (space) conditions have been reported to induce more biologically relevant 3D architecture in cell cultures,^[^ ^381^, ^382^ ^]^ and a variety of new mechanisms and gene expressions have been identified in cell cultures subjected to microgravitational forces.^[^ ^382^, ^383^ ^]^ Microgravity has also been shown to suppress tumor growth,^[^ ^384^ ^]^ which can be investigated in CoCs to understand the mechanism of tumor growth suppression and to possibly identify new anti‐cancer agents for the development of targeted chemotherapeutics. While CoCs have yet to be investigated under microgravitational conditions, future studies in this field could help identify potential early cancer targets, which can be used to design targeted cancer therapies for early‐stage treatment.^[^ ^381^, ^384^, ^385^ ^]^ Furthermore, excessive radiation exposure in space predisposes astronauts to cancer.^[^ ^382^ ^]^ OoCs and CoCs can be used for in‐depth investigation of the impact of space radiation on healthy and cancerous cells, to subsequently identify target mechanisms, genes, and biomolecules responsible for cancer predisposition. Targeted cancer therapeutics can then be designed for early prevention and/or treatment of cancer in astronauts and tested using CoCs. Recent studies have sent different OoCs to space to investigate the impact of space radiation, microgravity, and other conditions on these organs for the development of preventative treatments,^[^ ^386^ ^]^ including treatments for bone loss.^[^ ^387^ ^]^ In 2018, the “Tissue Chips in Space” project was launched in collaboration with NASA, the National Center for Advancing Translational Sciences (NCATS), the National Institutes of Health (NIH), and the Center for the Advancement of Science in Space (CASIS), which aimed to understand the impact of microgravity on human health by sending tissue chips, like OoCs, to space.^[^ ^386^ ^]^ Similarly, the Wyss Institute for Biologically Inspired Engineering launched the “Human Organ Chips for Radiation Countermeasure Development” project, funded by the FDA and supported by partnerships with NIH, Biomedical Advanced Research and Development Authority (BARDA), National Institute of Allergy and Infectious Diseases (NIAID), and the Division of Microbiology and Infectious Diseases at the Office of Biodefense Research Resources, and Translational Research.^[^ ^388^ ^]^ The progress of these projects is very promising, and future research combining space medicine, gravitational biology, and CoCs can open a variety of areas for the discovery of new, efficient, targeted cancer therapies.

## 7 Conclusions

Advances in microfluidic technology led to the development of novel, cancer‐on‐a‐chip in vitro platforms that have relatively high accuracy in recapitulating in vivo human cancer microenvironments, as compared to traditional 2D culture and animal studies. These platforms demonstrate great potential for use in developing and testing targeted chemotherapies of different targeting modalities, including passive, active, and triggered, to expedite bench‐to‐bedside translation. The use of CoC platforms in targeting studies remains relatively new and the advantages of CoCs in expediting and providing greater insight into targeted chemotherapeutic studies remain to be discovered. Nonetheless, in light of the advantages and disadvantages discussed above, the future directions of CoC application targeting studies are broad and promising. CoCs can provide an alternative testing modality that can augment or largely replace animal studies and help to design and select patients for more efficient clinical studies. CoCs would place as a significant base for the future of personalized medicine and targeted cancer therapies.

## Conflict of Interest

The authors declare no conflict of interest.
